# Unicore Enables Scalable and Accurate Phylogenetic Reconstruction with Structural Core Genes

**DOI:** 10.1093/gbe/evaf109

**Published:** 2025-06-02

**Authors:** Dongwook Kim, Sukhwan Park, Martin Steinegger

**Affiliations:** Interdisciplinary Program in Bioinformatics, Seoul National University, Seoul 08826, Republic of Korea; School of Biological Sciences, Seoul National University, Seoul 08826, Republic of Korea; Interdisciplinary Program in Bioinformatics, Seoul National University, Seoul 08826, Republic of Korea; School of Biological Sciences, Seoul National University, Seoul 08826, Republic of Korea; Interdisciplinary Program in Bioinformatics, Seoul National University, Seoul 08826, Republic of Korea; School of Biological Sciences, Seoul National University, Seoul 08826, Republic of Korea; Institute of Molecular Biology and Genetics, Seoul National University, Seoul 08826, Republic of Korea; Artificial Intelligence Institute, Seoul National University, Seoul 08826, Republic of Korea

**Keywords:** protein structure, core genes, phylogenetics, phylogenomics, bioinformatics

## Abstract

The analysis of single-copy core genes, common to most members of a clade, is important for key tasks in biology including phylogenetic reconstruction and assessing genome quality. Core genes are traditionally identified by the analysis of amino acid similarities among proteomes, but can also be defined using structures, which bear potential in deep clades beyond the twilight zone of amino acid sequence identity. Despite breakthroughs in accurate AI-driven protein structure prediction, obtaining full 3D structural models on a proteomic scale is still prohibitively slow. Here, we present Unicore, a novel method for identifying structural core genes at a scale suitable for downstream phylogenetic analysis. By applying the ProstT5 protein language model to the input proteomes to obtain their 3Di structural strings, Unicore saves over three orders of magnitude in runtime compared to a full 3D prediction. Using Foldseek clustering, Unicore identifies single-copy structures universally present in the species and aligns them using FoldMason. These structural core gene alignments are projected back to amino acid information for downstream phylogenetic analysis. We demonstrate that this approach defines core genes with linear run-time scaling over the number of species, up to seven times faster than OrthoFinder, while reconstructing phylogenetic relationships congruent with conventional approaches. Unicore is universally applicable to any given set of taxa, even spanning superkingdoms and overcoming limitations of previous methods requiring orthologs of fixed taxonomic scope and is available as a free and open source software at https://github.com/steineggerlab/unicore.

**Significance**

While the identification of core genes is at the heart of phylogenetic analysis, it has yet to benefit from the integration of structural information. Current approaches for structural core gene identification are constrained by computational inefficiencies, limiting their applicability for large-scale analyses. Unicore addresses this by offering a novel, scalable method that leverages 3Di structural strings computed by ProstT5, enabling fast and consistent identification of structural core genes, which in turn contribute to robust phylogenetic reconstruction. This advancement makes large-scale structure-informed phylogenetic analysis feasible and universally applicable, expanding the utilization of structural data in evolutionary biology.

## Introduction

Understanding the evolutionary relationships among organisms through phylogenetic analysis is a fundamental goal in biology. Phylogenomics allows us to reconstruct the tree of life, shedding light on how different species are related and how they have diverged over time (Eisen and Fraser 2003). A common phylogenomic approach leverages single-copy core genes—universally conserved across taxa—as reliable markers for reconstructing phylogenetic relationships (Rokas et al. 2003).

State-of-the-art core gene databases, including OrthoDB (Kriventseva et al. 2019), OMA (Altenhoff et al. 2024), UBCG (Kim et al. 2021), or UFCG (Kim et al. 2023), offer lists of single-copy core genes for specific clades. These gene sets are detected from genomes through sequence-based orthology inference (Altenhoff et al. 2019) or profile hidden Markov model (HMM)-based homology searches (Johnson et al. 2010). However, HMM- or all-vs-all search-based (Emms and Kelly 2019) approaches are computationally intensive and less effective for newly discovered clades, particularly when they are in the “twilight zone” from the known clades, where amino acid similarity is too low to detect homology (Rost 1999; Bordin et al. 2023). Protein structure-based methods, like Foldseek (van Kempen et al. 2024) using the 3Di alphabet, overcome these challenges by detecting homologs below the twilight zone through structural searches (Subbiah et al. 1993; Chung and Subbiah 1996; Bordin et al. 2023). Furthermore, Foldseek’s linear-scaling time clustering enables rapid proteome-wide comparisons (Steinegger and Söding 2018; Barrio-Hernandez et al. 2023).

The advent of AlphaFold2 (Jumper et al. 2021) has opened the door for much broader use of structural information in phylogenetic analysis by lifting the dependency on limitedly available experimentally determined structures (Andorf et al. 2022; Ruperti et al. 2023). Recent research shows that Foldseek’s 3Di alignments reveal deep evolutionary relationships and robust phylogenetic signals even for highly divergent sequences (Moi et al. 2023; Puente-Lelievre et al. 2023; Mifsud et al. 2024). However, applying AlphaFold2 or similar methods to even dozens of full proteomes for core gene detection would require months, limiting its use. Alternatively, protein language models (pLMs; Elnaggar et al. 2021; Meier et al. 2021; Lin et al. 2023) can be used to extract structural features directly from primary amino acid sequences, eliminating the need for computationally intensive structure prediction methods. Among them, ProstT5 (Heinzinger et al. 2024) stands out, which is a bilingual pLM trained on millions of sequence–structure pairs from the AFDB (Varadi et al. 2024). ProstT5 rapidly converts amino acid sequences into 3Di strings 3,600-fold faster than AlphaFold2-ColabFold (Mirdita et al. 2022), thereby making structure-informed large-scale phylogenetic analysis feasible.

**Fig. 1.**
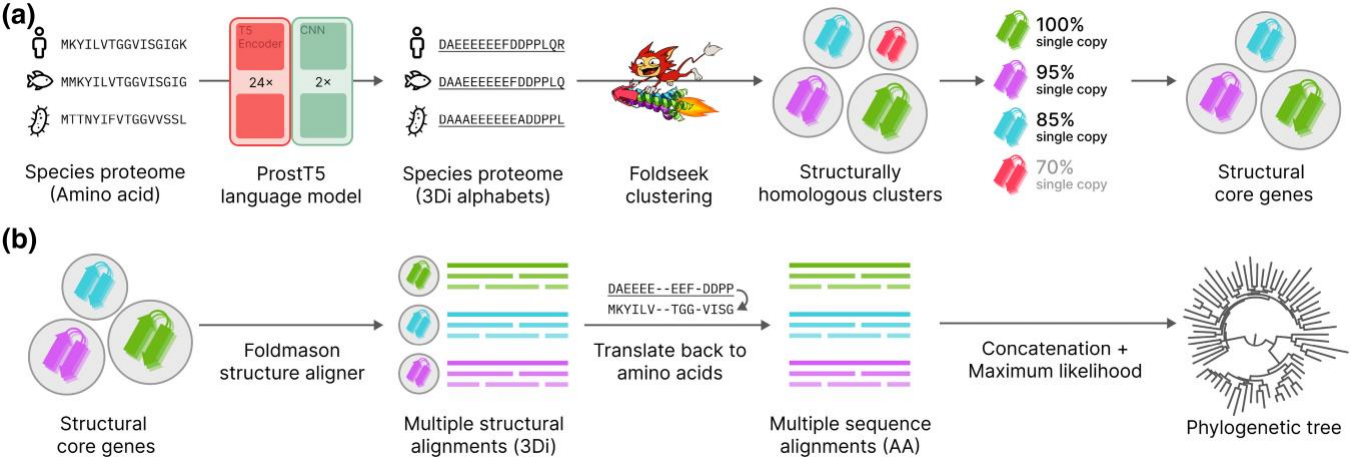
Graphical illustration of the Unicore workflow. a) The input species proteome, represented as amino acid sequences, is translated to 3Di alphabets (denoted by underscores) using the ProstT5 language model. These 3Di sequences are clustered with Foldseek to group structurally homologous proteins. Structural core genes are identified from these clusters by selecting those conserved as a single-copy in more than a specified proportion of the input species. b) FoldMason is used to construct MSTAs for each structural core gene cluster. These alignments are then converted back into amino acid sequences, enabling conventional evolutionary model-based maximum likelihood phylogenetic inference. Finally, a species phylogenetic tree is generated from the concatenated MSAs.

Here, we present Unicore, a scalable and accurate method for structure-based core gene definition and phylogenetic inference. Unicore leverages predicted 3Di sequences from the ProstT5 model and linear-time comparison methods (Steinegger and Söding 2018) to accelerate large-scale proteome analysis. It identifies single-copy “structural core genes” on the fly without relying on precomputed gene sets (Fig. 1). By integrating Foldseek’s rapid structural comparisons, Unicore identifies conserved structural genes across proteomes, generates structural alignments from 3Di strings using FoldMason (Gilchrist et al. 2024), and infers phylogenetic trees with maximum likelihood methods, such as IQ-TREE (Minh et al. 2020), FastTree (Price et al. 2010), and RAxML-NG (Kozlov et al. 2019).

## Results and Discussion

### Development of the Unicore Pipeline

We developed Unicore, a modular bioinformatics pipeline that defines structure-based core genes from species proteomes and reconstructs phylogeny based on their structural alignments. Its modules: createdb, cluster, profile, and tree are described below in detail. For ease of use and automated tree inference, easy-core orchestrates these modules into a single workflow.

Unicore createdb module takes a collection of species as an input, where each species is represented as a FASTA file of its proteome (Fig. 1a). These are translated into Foldseek’s 3Di alphabet (van Kempen et al. 2024) through the ProstT5 encoder-CNN (Heinzinger et al. 2024). Both the 3Di sequences and their corresponding amino acid sequences are compiled together into a Foldseek database. The cluster module then clusters the database with cascaded Foldseek clustering with reassignment, resulting in structural gene clusters. For each cluster, the profile module computes the fraction of species out of all input species that have exactly one member structure in that cluster. We denote this fraction as the single-copy coverage of the cluster. Clusters with coverage exceeding a certain threshold (in this study, 80% was used as a default value) are defined as the structural core genes.

From each structural core gene cluster, the tree module converts single-copy members into a FoldMason (Gilchrist et al. 2024) multiple structural alignment (MSTA; Fig. 1b). Gappy columns, defined as those with a gap fraction of 50% or higher, are then removed from the alignment. Finally, the remaining columns are converted back to amino acid sequences by mapping each 3Di alphabet to its corresponding residue, resulting in multiple sequence alignments (MSAs). From these structurally aligned MSAs, Unicore reconstructs the phylogeny by running either IQ-TREE, FastTree, or RAxML-NG (Price et al. 2010; Kozlov et al. 2019; Minh et al. 2020), where users can provide custom parameters for the tree builder as an argument to the --tree-options option. Unicore produces the maximum likelihood tree inferred from the concatenated MSAs of all identified structural core genes, as well as individual gene trees.

### Phylogenetic Reconstruction with Structurally Conserved Core Genes

**Fig. 2.**
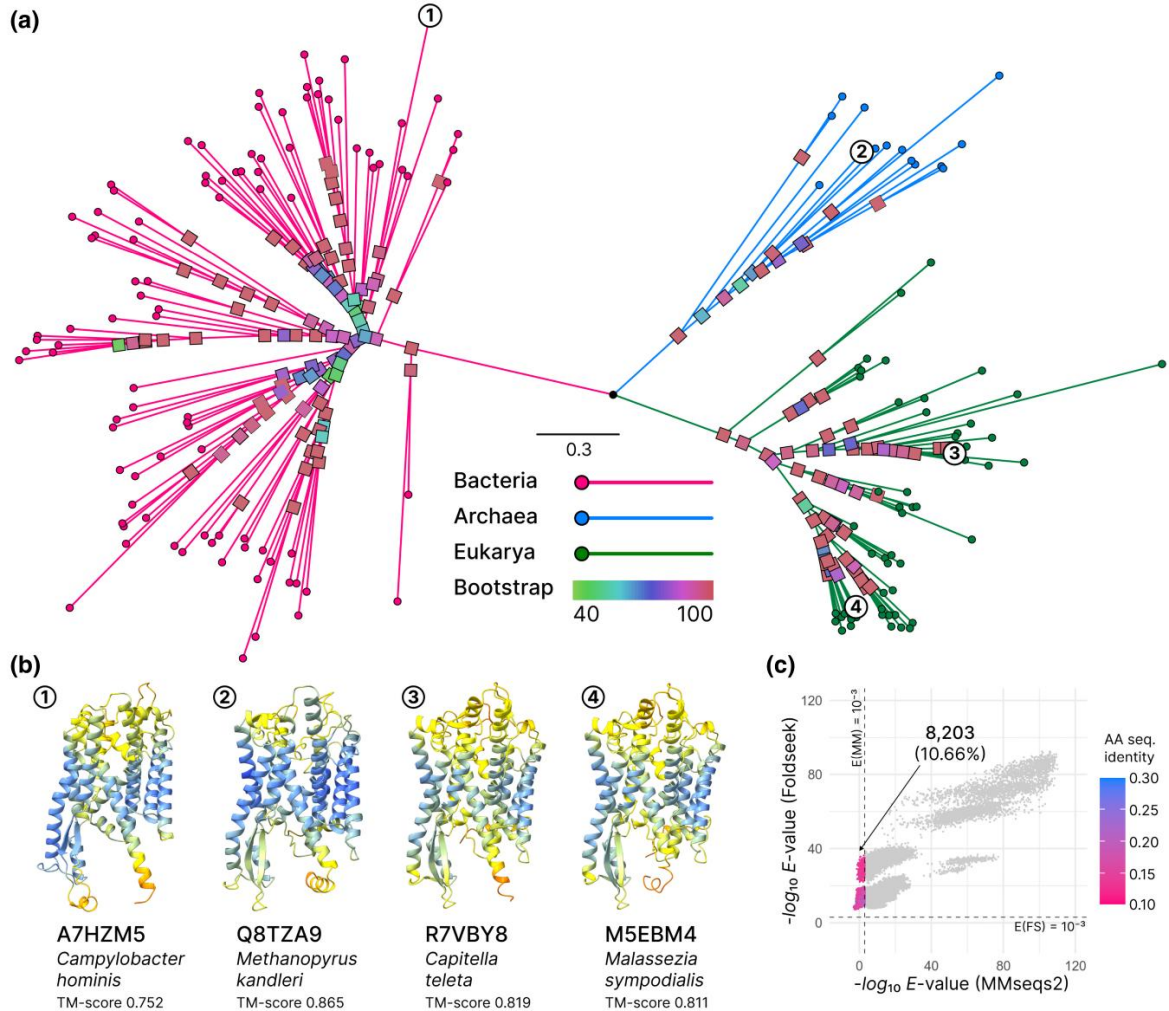
Structural core genes resolve phylogenetic relationships between species with distantly homologous proteins. a) Phylogenetic tree constructed from structural core genes identified across 166 species, spanning bacteria, archaea, and eukarya. Internal nodes are annotated with their bootstrap support values. b) Structural conservation illustrated by SecY protein translocase, one of the identified core genes. Predicted structural models are shown for four species, highlighting structural homology across bacteria (*Campylobacter hominis*), archaea (*Methanopyrus kandleri*), fungi (*Malassezia sympodialis*), and animalia (*Capitella teleta*), annotated with the TM-score from the alignment against PDB entry 1 rhz chain A. c) Scatter plot comparing *E*-values derived from MMseqs2 and Foldseek alignments of sequentially distant protein pairs (<30% sequence identity). The data points represent an all-against-all comparison from eight structural core genes uniquely defined by Unicore, which were not detected by OrthoFinder in the same species set. The dotted lines denote *E*(MMseqs) $=10^{-3}$ and *E*(Foldseek) $=10^{-3}$. The count and proportion of the partition with *E*(MMseqs) $>10^{-3}$ and *E*(Foldseek) $<10^{-3}$ are reported.

We identified 13 single-copy structural core genes with Unicore’s easy-core module (supplementary table 1, Supplementary Material online), shared by at least 80% out of 166 species spanning the three domains of life (see Materials and Methods). Phylogenetic inference using these structural core genes demonstrated monophyletic delineation of each of the three domains, supported by a bootstrap average of 91.83 (Fig. 2a, see supplementary fig. 1, Supplementary Material online for the detailed tree including species labels). An example of the structural conservation that characterizes Unicore’s core genes is the protein translocase subunit SecY (see supplementary table 1, Supplementary Material online for details), which was not identified as a core gene by OrthoFinder. SecY’s structures are conserved across archaea, bacteria, and eukarya (Fig. 2b) and in each case, predicted 3Di string aligns well with the experimentally defined structure (TM-score >0.5).

In cases where the taxonomic group of interest contains species with reduced or unconventionally structured genomes, such as parasitic or symbiotic species that may undergo reductive evolution (Andersson and Kurland 1998; Albalat and Cañestro 2016), can impact the definition of the core genes and subsequent phylogenetic analysis. To prevent this, the user receives a warning if any of their input proteomes fail to represent the majority of the defined set of core genes.

Next, we assessed the utility of structural comparison beyond the twilight zone by comparing *E*-values between MMseqs2’s amino acid and Foldseek’s 3Di alignments of structural core genes (Fig. 2c). Foldseek rescued 8,203 homologous pairs that yielded amino acid alignments with lower confidence (*E*-value $>10^{-3}$) by structurally aligning them, underpinning the capability of 3Di alignments in elucidating distant homology for phylogenetics (Moi et al. 2023; Puente-Lelievre et al. 2023; Mifsud et al. 2024).

Here, we applied an 80% threshold for single-copy species coverage to ensure a sufficient number of genes are found to conduct downstream phylogenetic analyses. Adjusting this coverage threshold can impact the resulting set of core genes, which inevitably affects the subsequent phylogenetic analysis (Kim et al. 2023). Therefore, we made this threshold customizable in Unicore, along with the parameters for Foldseek’s modules, allowing users flexibility in adjusting them according to their specific needs.

### Comparison Between Structural and Sequence-Based Core Genes

We evaluated Unicore’s core genes by comparing them with those identified by sequence-based methods, specifically OrthoFinder (Emms and Kelly 2019) and BUSCO (Manni et al. 2021). Out of Unicore’s 92,500 core genes identified in 163 bacterial and 98 fungal sets (see Materials and Methods), 83,700 (90.49%) were detected as such by both OrthoFinder and BUSCO (supplementary fig. 2a, Supplementary Material online), while 81 (0.09%) genes were unique to Unicore. In the opposite direction, we found 86.89% of OrthoFinder’s genes and 89.94% of BUSCO’s genes were detectable by Unicore (supplementary fig. 2b and c, Supplementary Material online), further supporting the validity of Unicore’s core genes.

We next examined the core genes uniquely defined by Unicore, i.e. those identified only through structural comparisons. Of the 81 unique core genes, 52 (64.20%) contained pairwise alignments with sequence identity below 30%, collectively forming 7,030 pairs beyond the twilight zone. Among them, 878 (12.49%) pairs could be rescued by Foldseek structural alignment (supplementary fig. 3, Supplementary Material online).

Despite the smaller gene set, 33 (40.74%) of the 81 structurally unique core genes were found in inter-phyla species sets (supplementary fig. 2d, Supplementary Material online). These sets showed significantly greater phylogenetic diversity (one-sided Mann–Whitney *U* test), measured by mean branch length, than those at lower taxonomic levels (supplementary fig. 4, Supplementary Material online). Both inter-phyla sets showed the highest proportion of structurally unique core genes (bacteria: 0.18%, fungi: 0.32%), and in fungi, these values increased as the taxonomic level gets broader. These findings, aligned with Unicore’s performance at the scale of the tree of life, suggest that Unicore’s ability to detect remote homology improves with the phylogenetic diversity of the input.

We then delved into the 80,302 core genes of OrthoFinder (70,821 genes) and BUSCO (9,481 genes) that Unicore failed to identify. Among these, 39,005 (48.57%) were mapped to the foldseek clusters (i.e. Unicore core gene candidates) by sharing more than 80% of their members. However, Unicore excluded 32,446 (83.18%) of them due to the presence of excessive multiple copies (supplementary fig. 5, Supplementary Material online). We speculate this is primarily due to the methodological differences, where OrthoFinder and BUSCO identify core genes through orthology based on reciprocal best hits, while Unicore uses a sensitive and transitive clustering method that can be more permissive in assigning genes as multiple copies.

Additionally, out of 41,297 genes left unmapped, 23,821 (57.68%) were found as fragments across multiple foldseek clusters due to Unicore’s strict alignment coverage threshold, which requires covering at least 80% for both query and target proteins. While OrthoFinder further adjusts the alignment bit scores by sequence length, our strict coverage criteria introduce missing links between homologs with length discrepancies, eventually rejecting them as core genes. As a trade-off, however, our method produces a more specific, condensed set of core genes with enhanced scalability, offering computational advantages in the downstream phylogenetic analysis.

### Robustness of the Phylogenetic Trees Reconstructed with Unicore

**Fig. 3.**
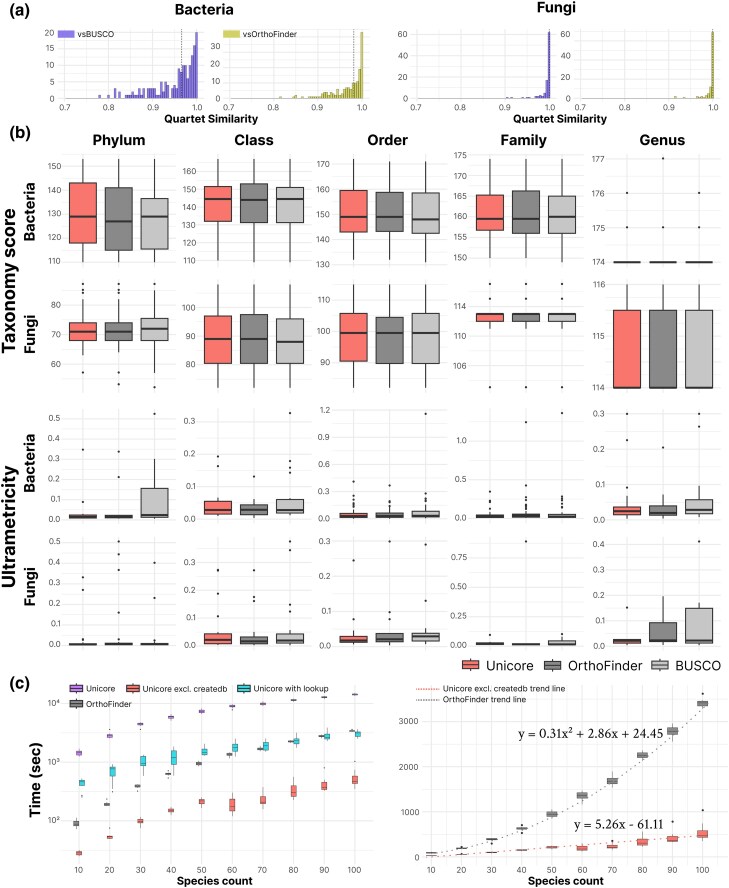
Benchmark results comparing the speed and phylogenetic robustness of trees generated by Unicore, OrthoFinder, and BUSCO. a) The histogram shows quartet similarity scores for trees computed with Unicore against BUSCO and OrthoFinder. The left panel displays bacterial trees, while the right shows fungal trees. A bin width of 0.005 is used, with the median marked by a vertical dotted line. b) TCS and ultrametricity for Unicore (left), OrthoFinder (middle), and BUSCO (right) are compared across taxonomic ranks. c) Left: Unicore includes the entire process from predicting 3Di sequences to core gene inference. Unicore with lookup accounts only for predicting 3Di sequences absent in the AlphaFold database, while Unicore excl. createdb benchmarks core gene inference alone. OrthoFinder measures orthogroup inference. Right: Results are plotted on a linear y-axis. Core gene inference with Unicore exhibits linear time complexity, unlike OrthoFinder’s quadratic scaling, as shown by the dotted trend line. Regression analysis confirmed this, with Unicore fitting better to a linear model (linear coef = 5.27, $p<10^{-15}$) than to a quadratic model (quadratic coef = 0.045, p = 0.0007). In contrast, OrthoFinder’s runtime fits well with a quadratic model (quadratic coef = 0.3095, $p<10^{-15}$).

We further assessed the quality of phylogenetic inference with Unicore structural core genes identified in the 163 bacterial and 98 fungal benchmark sets. Unicore’s bacterial trees showed median quartet similarity to OrthoFinder’s and BUSCO’s trees was 0.984 and 0.969, respectively (Fig. 3a, left). This measure was even higher in the fungal dataset, reaching 1.0 compared to both methods (Fig. 3a, right). However, this may result from having smaller fungal sets than bacterial sets (see Materials and Methods), an artifact due to the limited availability of high-quality fungal genomes.

Across all taxonomic ranks for bacteria and fungi, Unicore trees exhibited comparable taxonomic congruence (TCS) and ultrametricity to those based on core genes from OrthoFinder and BUSCO (Fig. 3b). The indistinction of ultrametricities between Unicore and sequence-based methods is notable, since Moi et al. (2023) recently reported in their method, FoldTree, that their structure-based phylogenetic trees had yielded lower ultrametricity, implying better representation of the molecular clock. However, such a discrepancy is likely due to the fundamental difference between the methodologies of Unicore and FoldTree. In FoldTree, they utilized a highly diverse dataset to build gene trees with the neighbor-joining algorithm. Contrastly, we employed proteomes spanning across phyla at most in this benchmark, with the purpose of generating species trees with the maximum likelihood method. By the difference in the type of phylogenetic tree they aim to compute, we also could not conduct a benchmark directly comparing Unicore against FoldTree.

To evaluate potential benefits of structure-based alignment, we compared phylogenetic trees generated from MSAs produced by FoldMason (structure-based) and MAFFT-linsi (sequence-based). We observed no significant differences between the resulting trees (supplementary fig. 6, Supplementary Material online), likely due to the robust inference capability of the supermatrix approach. Nevertheless, FoldMason remains advantageous for constructing MSAs as it offers faster and more accurate alignment compared to traditional sequence-based aligners (Gilchrist et al. 2024).

Throughout this section, we applied the same evolutionary model across all concatenated MSAs to enable direct comparison. For those requiring phylogenetic inferences with complex models, we provide a partition file in RAxML-style format that users can easily modify and forward to IQ-TREE or RAxML-NG.

Put together, structural core genes of Unicore reconstructed robust species phylogeny comparable to the sequence-only methods, highlighting both the validity of the structure-based phylogenetic approach and its prospect of elucidating beyond the twilight zone of amino acid sequences.

### Unicore Allows Structure-Based Phylogeny at Scale

To demonstrate the scalability of our method, we compared the time it takes Unicore and OrthoFinder to detect core genes in benchmark sets of species. Excluding database creation, Unicore scales linearly with the number of input species, thereby being faster than the quadratically scaling OrthoFinder, reaching a factor of 6.92 in runtime over 100 species (Fig. 3c). However, database creation is Unicore’s biggest bottleneck, which requires 3Di conversion by ProstT5, making it in total up to 4.19 times slower than OrthoFinder. To alleviate this, we implemented a sequence lookup method that rapidly retrieves 3Di sequences from the AFDB (Varadi et al. 2024) for exact sequence matches, thereby improving Unicore’s runtime by up to 4.77-fold. Additionally, createdb module accepts precomputed Foldseek database via --custom-lookup option, allowing for bypassing the 3Di conversion in cases where the protein structures of input species are readily present.

Since conversion with ProstT5 heavily depends on the model and amount of GPU resources, the overall throughput is expected to be improved concurrently with the rapid development of graphics hardware. In this context, owing to its quasilinear clustering algorithm (Steinegger and Söding 2018), Unicore’s scalability will become increasingly powerful against the ever-increasing flood of biological data.

### Concluding Remarks

Unicore implements a large-scale, structure-based phylogenetic method powered by state-of-the-art tools for protein structural analysis, compiled into a fully automated bioinformatics pipeline. Accompanied by the recent developments in structural phylogenetics (Moi et al. 2023; Puente-Lelievre et al. 2023; Garg and Hochberg 2024; Gilchrist et al. 2024; Mifsud et al. 2024), we anticipate our tool to further expand the utility of protein structures from an evolutionary perspective.

## Materials and Methods

### Tree of Life-Wide Phylogeny Using Unicore

We gathered the reference proteome from the UniProt release 2024_02 (The UniProt Consortium 2023), filtered those with >1,000 proteins and containing >90% of single-copy BUSCOs (v5.4.7) with respective domain used as an OrthoDB v10 lineage (Kriventseva et al. 2019), resulting in 9,163 proteomes. We then randomly sampled one species from each class, resulting in 166 species comprising all of the bacterial, archaeal, and eukaryotic domains (supplementary table 2, Supplementary Material online). We ran Unicore v1.1.0 easy-core module on these species with default parameters to produce a structural core gene tree with Foldseek v10-941cd33, FoldMason v1-763a428, and IQ-TREE v2.2.2.3. Protein structures were visualized using UCSF ChimeraX v1.9 (Meng et al. 2023).

### Consistency Benchmark—Preparation of Species Sets

Starting with the 9,163 quality-controlled proteomes from the tree of life analysis, we identified 188 taxa ranging from genus to phylum as having at least *N* member species (using N=30 for bacteria and N=20 for fungi), according to NCBI taxonomy annotations (Schoch et al. 2020). We randomly divided each of these 188 taxa into sets of proteomes with exactly *N* members (e.g. fungal taxa with 41 species would yield two sets of 20 and one left out proteome), resulting in 876 bacterial and 98 fungal species sets (supplementary table 3, Supplementary Material online). Of these, we randomly sampled one set from each bacterial taxa, resulting in 163 bacterial sets, which were used for the following analyses along with the entirety of 98 fungal sets (supplementary table 4, Supplementary Material online).

### Consistency Benchmark—Comparison with Sequence-Based Methods

We computed structure- and sequence-based core genes from each of the 163 and 98 proteome sets using Unicore easy-core and OrthoFinder (v2.5.5), represented as single-copy genes in at least 90% of the species within each set. This is more stringent than the default threshold, applied to ensure the independent comparison between the tools in terms of the species coverage threshold. To this end, only the first three modules of Unicore were run with default parameters, except for profile, which was run with the option -t 0.9. For OrthoFinder, we used the arguments -og -S mmseqs and extracted orthogroups that cover at least 90% of the species within the dataset. Additionally, BUSCO v5.4.7 with default parameters was used to extract the respective lineage of OrthoDB v10 core genes from each set.

For the core genes defined by each method, we performed HMM-HMM comparisons using hhsearch in HH-suite3 v3.3.0 (Steinegger et al. 2019) with MAFFT-generated MSAs as input (Katoh and Standley 2013). Each MSA was compared against core gene sets from other methods, with a pair classified as shared if the global alignment yielded an *E*-value $<10^{-3}$ with at least 80% coverage. Based on these comparisons, each core gene was classified as shared by both methods or as unique to a specific method.

To investigate the core genes missed by Unicore, we first filtered the genes down to those that can be mapped to the Foldseek clusters (i.e. Unicore core gene candidates), by accepting those that share at least 80% of their members with a single Foldseek cluster. For each of the mapped Foldseek clusters, we obtained the ratio of species covered by single-copied genes and multiple-copied genes. We then categorized the clusters by 90% single-copy species coverage, as well as by 90% coverage combining both single- and multiple-copied genes.

For the remaining genes that were unmapped to the Foldseek clusters, we assessed whether they were unidentified due to the strict alignment coverage threshold we applied. For this, we first structurally aligned the representatives of the Foldseek clusters against themselves using Foldseek search with default parameters. For each remaining core gene, we defined that the gene was fragmented across multiple Foldseek clusters if the representative sequences of the two clusters sharing the largest number of members with this gene yielded a Foldseek alignment (*E*-value $<10^{-3}$) with an alignment coverage below 0.8.

### Assessment on Phylogenetic Robustness

We utilized the Unicore, OrthoFinder, and BUSCO core genes of the benchmark species sets from the “Consistency benchmark” section to compare the phylogenetic trees reconstructed from each tool. Here, we adjusted the single-copy threshold of Unicore and OrthoFinder to their default values—80% and 100%, respectively—to obtain a computationally suitable amount of core genes for the phylogenetic analysis. We used the tree module with default parameters to generate phylogenetic trees from the concatenated alignments of Unicore core genes. For the core genes of OrthoFinder and BUSCO, we computed MSAs using MAFFT-linsi v7.525 (Katoh and Standley 2013) with default settings, filtered out columns with over 50% gaps, and constructed phylogenetic trees using IQ-TREE v2.3.6 (Minh et al. 2020) with parameters -m JTT+F+I+G -B 1000 on the concatenated filtered MSAs. All resulting phylogenetic trees were rooted to yield minimum ancestor deviation (Tria et al. 2017).

First, we calculated quartet similarity between the trees with tqDist v1.0.2 (Estabrook et al. 1985; Sand et al. 2014). Then, we used the metrics introduced by FoldTree (Moi et al. 2023), namely taxonomic congruence scores (TCS) to the NCBI taxonomy (Schoch et al. 2020) and ultrametricity using FoldTree source code (commit 92fd2c3), to further benchmark the quality of the phylogenetic trees. We calculated the mean branch length of the phylogenetic trees with Gotree v0.4.3 (Lemoine and Gascuel 2021)

To test for statistical significance, we performed a *t*-test with the alternative hypothesis that Unicore trees exhibit either higher TCS or lower ultrametricity compared to those generated by OrthoFinder and BUSCO. We controlled for multiple tests by adjusting the *p*-values using the Benjamini–Hochberg method (Benjamini and Hochberg 1995) for comparisons with OrthoFinder and BUSCO independently.

### Computation Time Benchmark

We compared Unicore’s and OrthoFinder’s runtimes on sets of 10, 20, 30, …, 100 species. The species in these sets were randomly sampled from the 330 species of the 11 bacterial sets at the level “phyla” identified as described in the “Consistency benchmark” section (supplementary table 4, Supplementary Material online). For Unicore, we used createdb with --max-len 4000 -g to generate 3Di databases, cluster with the arguments -c “-c 0.8 --cluster-reassign 1 --max-seqs <maxseqs>”, where maxseqs is set to the larger of 1,000 or 20 times the number of species in the proteome database, and profile with default parameters. We separately estimated the time spent on 3Di conversion (createdb) and core gene definition (cluster + profile). Orthofinder was executed with the arguments -og -S mmseqs. We used one NVIDIA RTX 4,090 to run Unicore createdb, and 128 threads of AMD EPYC 7702P to define core genes using both tools, and took an average runtime of 10 repeated runs. For both tools, we fit a linear regression model between the number of species and the tool’s runtime using R v4.4.1.

## Supplementary Material

evaf109_Supplementary_Data

## Data Availability

Unicore is implemented in Rust and is available as GPLv3 licensed free open-source software at https://github.com/steineggerlab/unicore.
