# Supplementary material for: Unicore Enables Scalable and Accurate Phylogenetic Reconstruction with Structural Core Genes
Source: Genome Biol Evol. 2025 Jun 2;17(6):evaf109. doi: 10.1093/gbe/evaf109 (PMC12203212; doi:10.1093/gbe/evaf109)
Supplement: evaf109_Supplementary_Data [file evaf109_supplementary_data.pdf]

Unicore Enables Scalable and Accurate Phylogenetic Reconstruction with Structural Core Genes

Dongwook Kim [1,2,†], **Sukhwan Park** [1,2,†], **and Martin Steinegger** [1,2,3,4,*]

[1]Interdisciplinary Program in Bioinformatics, Seoul National University, Seoul 08826, Republic of Korea, [2]School of Biological Sciences, Seoul National University, Seoul 08826, Republic of Korea, [3]Institute of Molecular Biology and Genetics, Seoul National University, Seoul 08826, Republic of Korea, [4]Artificial Intelligence Institute, Seoul National University, Seoul 08826, Republic of Korea

SUPPLEMENTARY MATERIALS

Figures

- Supplementary Fig. 1. Structural core gene tree of 166 species across the tree of life.
- Supplementary Fig. 2. Venn diagram representation of the overlap between the orthologs.
- Supplementary Fig. 3. Comparison of MMseqs2 against Foldseek alignment *E*-values of 81 core genes uniquely defined by Unicore.
- Supplementary Fig. 4. Statistics of Unicore phylogenetic trees for the benchmark species sets.
- Supplementary Fig. 5. Distribution of Foldseek clusters with at least 80% overlap to core genes not identified by Unicore.
- Supplementary Fig. 6. Benchmark results comparing the phylogenetic robustness of trees generated from multiple sequence alignments produced by Foldmason and MAFFT-linsi.

Tables

- Supplementary Table 1. List of 13 structural core genes defined from 166 species spanning the tree of life.
- Supplementary Table 2. Taxonomic description of 166 species spanning across the tree of life.
- Supplementary Table 3. List of the 188 taxa and 974 sub-sampled sets of species defined from 9,163 quality-controlled proteomes.
- Supplementary Table 4. Detailed list of the 261 sets on which benchmark was performed.

*To whom correspondence should be addressed. Email: martin.steinegger@snu.ac.kr
†These authors contributed equally.

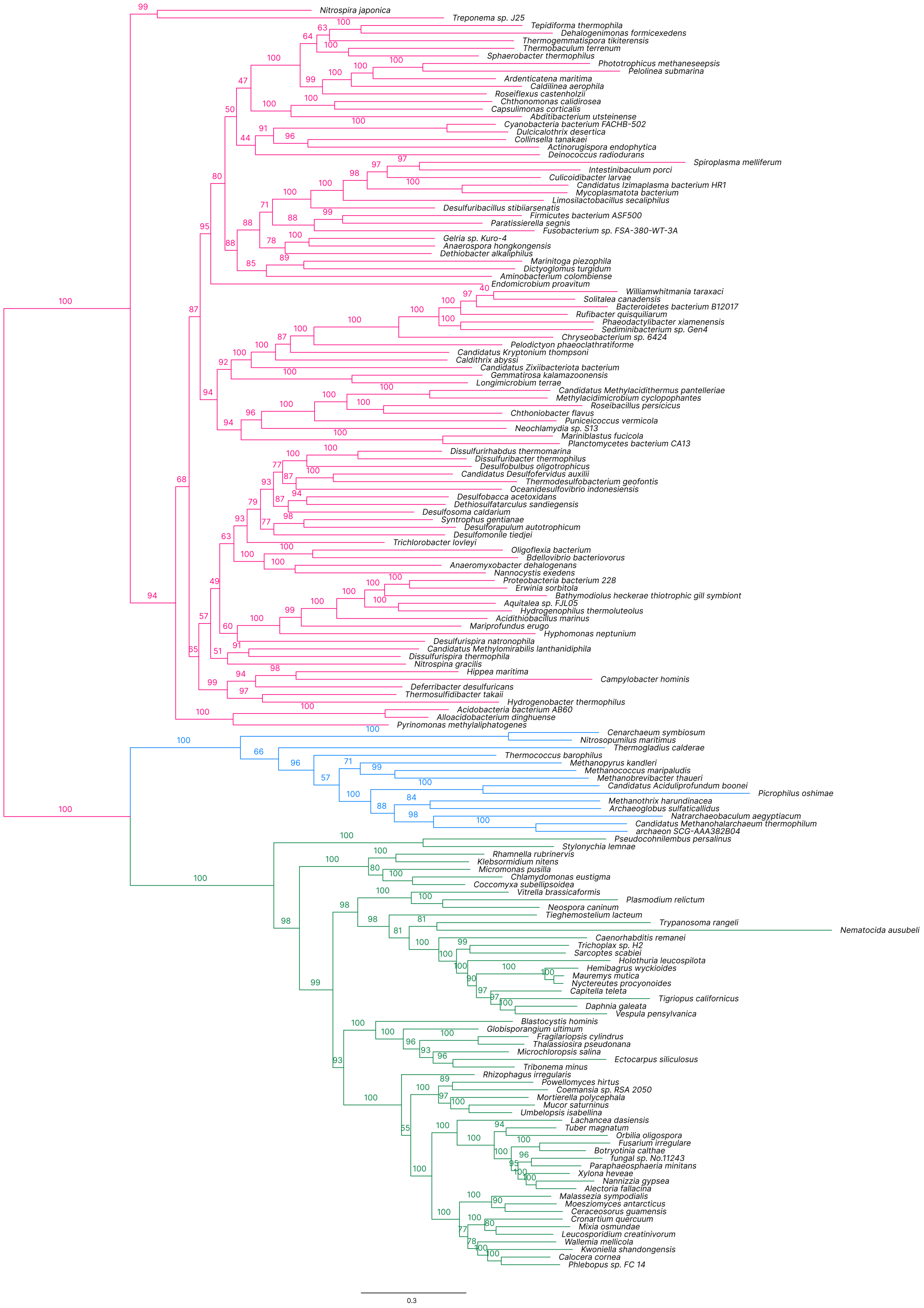

Supplementary Fig. 1. Structural core gene tree of 166 species across the tree of life. Branches and bootstrap support values are colored by their originating domain (magenta, Bacteria; cyan, Archaea; green, Eukarya).

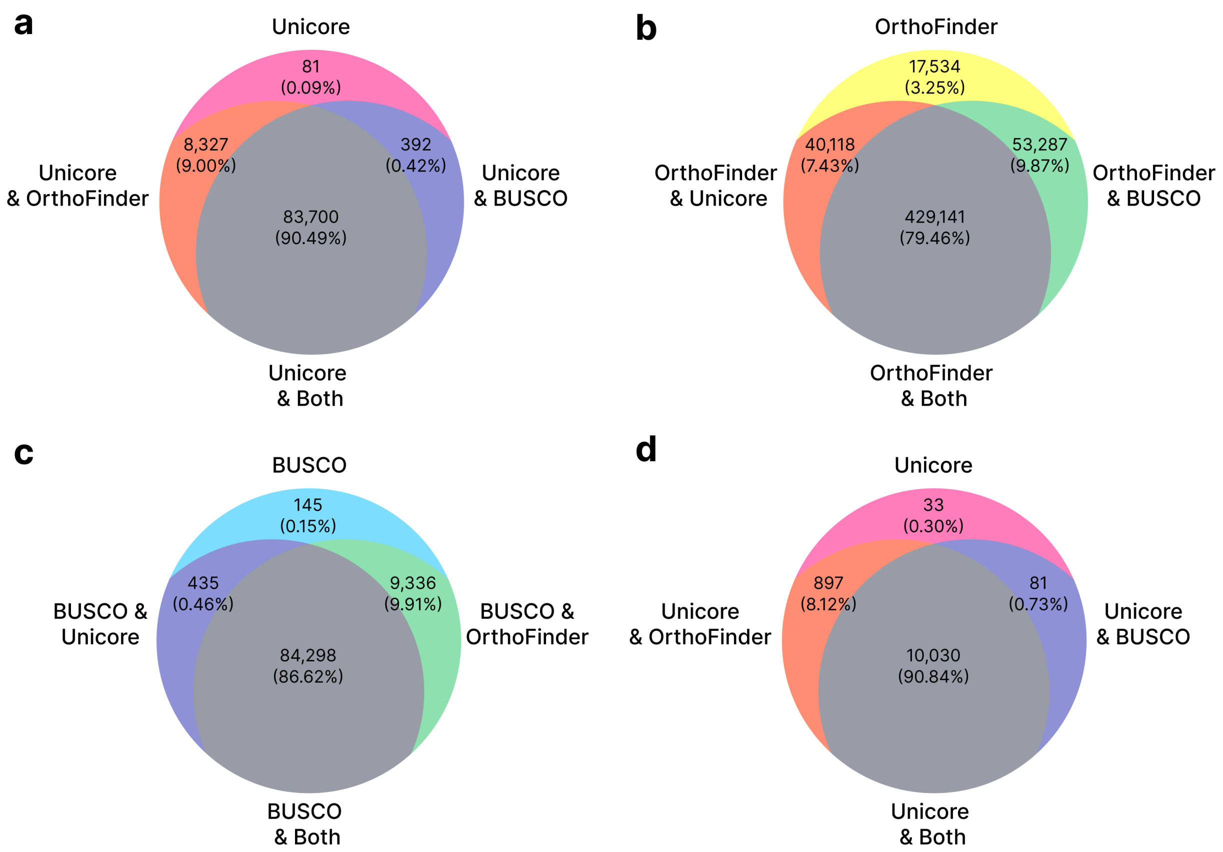

Supplementary Fig. 2. Venn diagram representation of the overlap between the orthologs. Ratios of the orthologs shared with none, either or both of the other tools to the total number of orthologs are shown for (a) Unicore, (b) OrthoFinder, (c) BUSCO, and (d) Unicore, subsampled with inter-phyla datasets.

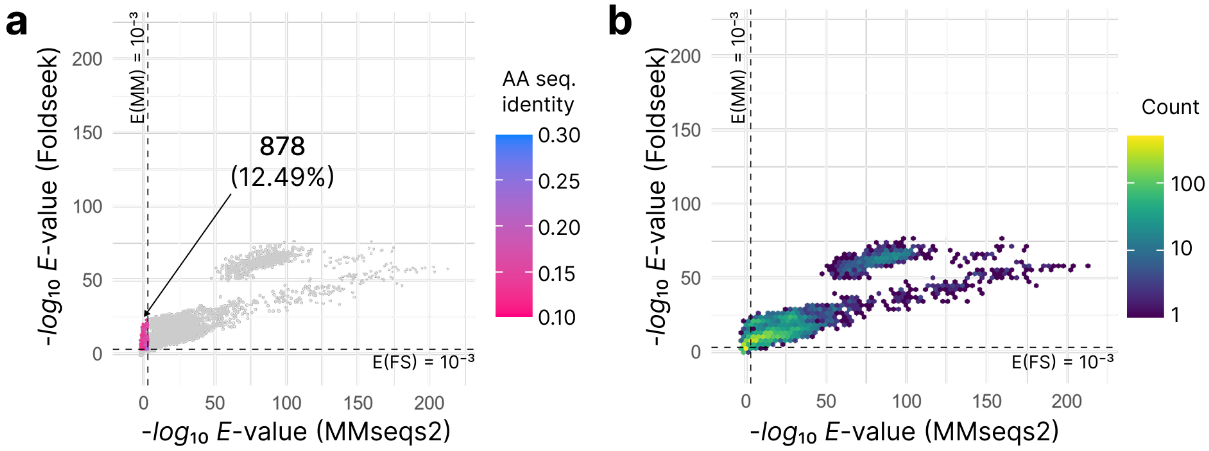

Supplementary Fig. 3. Comparison of MMseqs2 against Foldseek alignment E-values of 81 core genes uniquely defined by Unicore, visualized as (a) scatter plot and (b) density map of hexagonal bins. Visualization details are identical to those of Fig. 2c.

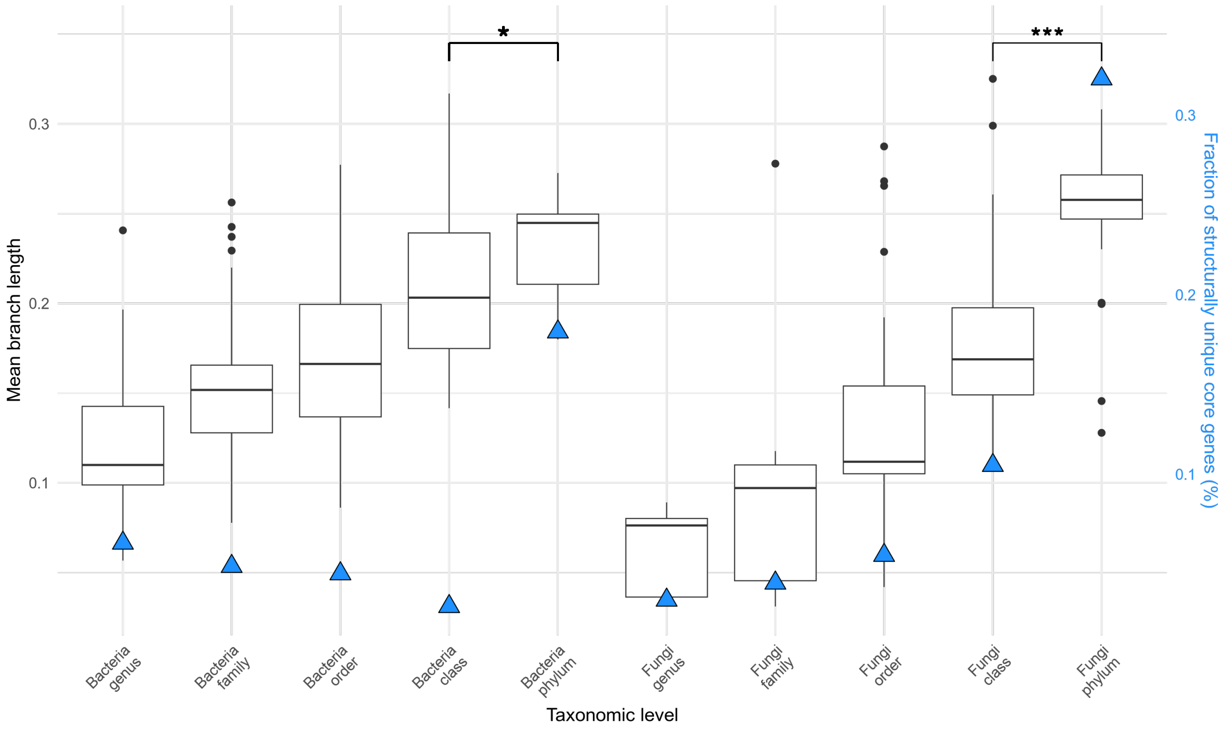

Supplementary Fig. 4. Statistics of Unicore phylogenetic trees for the benchmark species sets, grouped by taxonomic level. Mean branch lengths are shown as a box-and-whisker plot, where boxes represent the first, second, and third quartiles, and whiskers extend to 1.5× IQR. Outliers are shown as individual points. Statistical significances are annotated for two pairs of interest: Fungi phylum against Fungi class, and Bacteria phylum against Bacteria phylum (One-sided Mann-Whitney U-test, * $p < 0.05$; *** $p < 0.001$). Percentages of structurally unique core genes at each taxonomic level, shown as blue triangles, are overlaid on the boxplot.

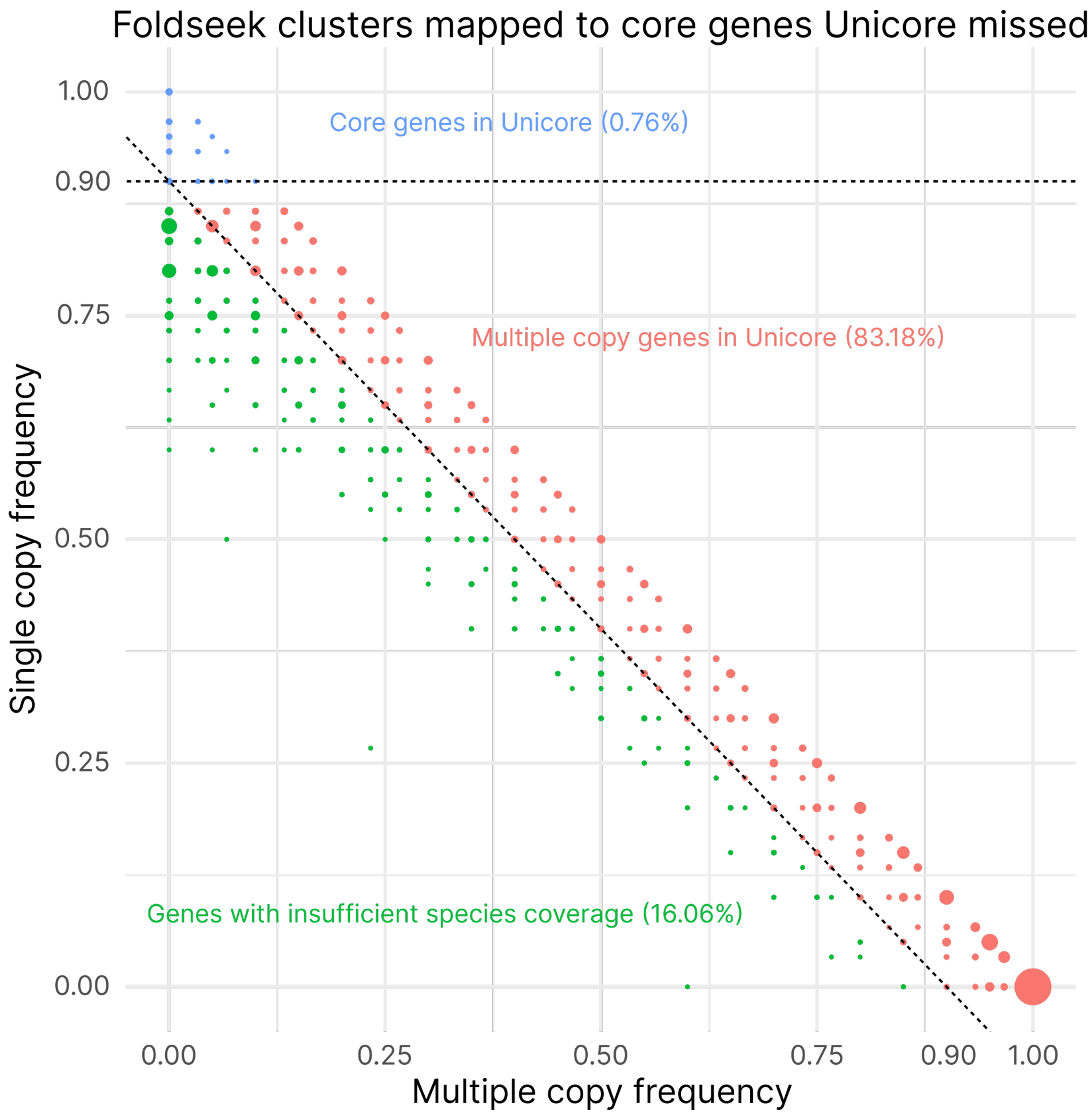

Supplementary Fig. 5. Distribution of Foldseek clusters with at least 80% overlap to core genes not identified by Unicore. The x-axis and y-axis indicate the fraction of species with multiple- and single-copy genes per cluster, respectively. Blue dots denote clusters assigned as core genes by Unicore; red dots indicate clusters not assigned as core genes due to containing multiple-copy genes; green dots represent clusters lacking sufficient species representation to be potential core genes. Dot size corresponds to the number of clusters sharing the same frequency of multiple- and single-copy genes.

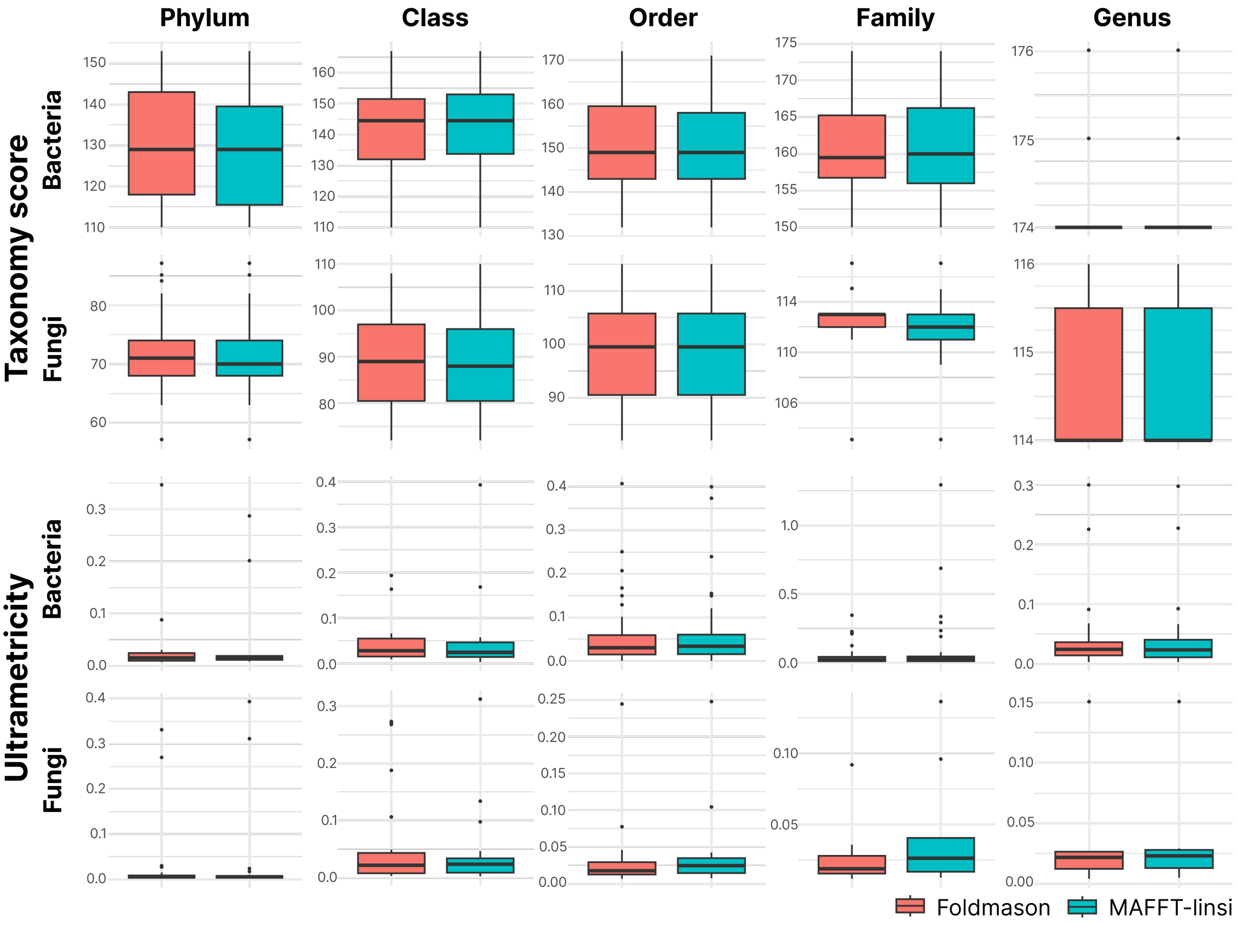

Supplementary Fig. 6. Benchmark results comparing the phylogenetic robustness of trees generated from multiple sequence alignments produced by Foldmason and MAFFT-linsi. TCS and ultrametricity for Foldmason (red) and MAFFT-linsi (teal) are compared across taxonomic ranks.

Supplementary Table 1. List of 13 structural core genes defined from 166 species spanning the tree of life. For each core gene, representative identifier and functional description annotated by UniProt are described, as well as overlapping status against OrthoFinder.

Identifier	Functional desciption	OrthoFinder
A0A196SAG3	Large ribosomal subunit protein L27	Found
A0A250X2L9	Glutamine-hydrolyzing GMP synthase	Found
A0A2S8SQG3	Phenylalanine--tRNA ligase subunit B	Not found
A0A6I6EN72	Small ribosomal subunit protein uS3	Not found
A0A811YLJ7	Large ribosomal subunit protein uL1	Not found
A0A811ZT41	Small ribosomal subunit protein uS12	Not found
A0A8J2WDH5	Small ribosomal subunit protein uS9	Not found
A0A8K0XC75	Protein translocase subunit SecY	Not found
A0A918WN48	Alanine--tRNA ligase	Found
A0A9D3NI02	Adenylosuccinate synthetase	Found
A0A9Q1BBL0	Large ribosomal subunit protein uL5	Not found
B8BRS9	Large ribosomal subunit protein uL11	Found
Q0BYC9	Large ribosomal subunit protein uL6	Not found

Supplementary Table 2. Taxonomic description of 166 species spanning across the tree of life. For each species we described its class, from which it was randomly selected among the members, along with its UniProt identifier of reference proteome.

Identifier	Species name	Class name
UP000000263	*Roseiflexus castenholzii*	Chloroflexia
UP000000323	*Thermobaculum terrenum*	Chloroflexota
UP000000442	*Desulforapulum autotrophicum*	Desulfobacteria
UP000000483	*Desulfobacca acetoxidans*	Desulfobaccia
UP000000590	*Methanococcus maripaludis*	Methanococci
UP000000758	*Cenarchaeum symbiosum*	Nitrososphaerota
UP000000792	*Nitrosopumilus maritimus*	Nitrososphaeria
UP000001400	*Aciduliprofundum boonei**	Thermoplasmatota*
UP000001449	*Thalassiosira pseudonana*	Coscinodiscophyceae
UP000001520	*Deferribacter desulfuricans*	Deferribacteres
UP000001826	*Methanopyrus kandleri*	Methanopyri
UP000001876	*Micromonas pusilla*	Mamiellophyceae
UP000001935	*Anaeromyxobacter dehalogenans*	Myxococcia
UP000001959	*Hyphomonas neptunium*	Alphaproteobacteria
UP000002027	*Sphaerobacter thermophilus*	Thermomicrobia
UP000002366	*Aminobacterium colombiense*	Synergistia
UP000002407	*Campylobacter hominis*	Epsilonproteobacteria
UP000002420	*Trichlorobacter lovleyi*	Desulfuromonadia
UP000002524	*Deinococcus radiodurans*	Deinococci
UP000002574	*Hydrogenobacter thermophilus*	Aquificae
UP000002630	*Ectocarpus siliculosus*	Phaeophyceae
UP000002669	*Nannizzia gypsea*	Eurotiomycetes
UP000002724	*Pelodictyon phaeoclathratiforme*	Chlorobiia
UP000004671	*Caldithrix abyssi*	Calditrichia
UP000004830	*Collinsella tanakaei*	Coriobacteriia
UP000005242	*Wallemia mellicola*	Wallemiomycetes
UP000005270	*Thermogladius calderae*	Thermoprotei
UP000005824	*Chthoniobacter flavus*	Spartobacteria
UP000005877	*Methanothrix harundinacea*	Methanomicrobia
UP000006055	*Desulfomonile tiedjei*	Desulfomonilia
UP000006443	*Dethiobacter alkaliphilus*	Dethiobacteria
UP000006583	*Thermodesulfobacterium geofontis*	Thermodesulfobacteria
UP000007161	*Marinitoga piezophila*	Thermotogae
UP000007264	*Coccomyxa subellipsoidea*	Trebouxiophyceae
UP000007478	*Thermococcus barophilus*	Thermococci
UP000007494	*Neospora caninum*	Conoidasida
UP000007590	*Solitalea canadensis*	Sphingobacteriia
UP000007719	*Dictyoglomus turgidum*	Dictyoglomia
UP000007880	*Caldilinea aerophila*	Caldilineae
UP000008139	*Hippea maritima*	Desulfurellia
UP000008281	*Caenorhabditis remanei*	Chromadorea

Supplementary Table 2. (Continued.)

Identifier	Species name	Class name
UP000008784	*Orbilia oligospora*	Orbiliomycetes
UP000009131	*Mixia osmundae*	Mixiomycetes
UP000011704	*Nitrospina gracilis*	Nitrospinia
UP000011976	*Moesziomyces antarcticus*	Ustilaginomycetes
UP000013307	*Archaeoglobus sulfaticallidus*	Archaeoglobi
UP000014227	*Chthonomonas calidirosea*	Chthonomonadetes
UP000014760	*Capitella teleta*	Polychaeta
UP000017395	*Firmicutes* bacterium ASF500*	Bacillota
UP000019132	*Globisporangium ultimum*	Oomycota
UP000019151	*Gemmatirosa kalamazoonensis*	Gemmatimonadetes
UP000029408	*Izimaplasma* bacterium HR1*	Izimaplasma*
UP000029736	*Phaeodactylibacter xiamenensis*	Saprospiria
UP000031518	*Pyrinomonas methylaliphatogenes*	Blastocatellia
UP000031737	*Trypanosoma rangeli*	Kinetoplastea
UP000032233	*Dethiosulfatarculus sandiegensis*	Desulfarculia
UP000035337	*Endomicrobium proavitum*	Endomicrobiia
UP000037784	*Ardenticatena maritima*	Ardenticatenia
UP000039865	*Stylonychia lemnae*	Spirotrichea
UP000041254	*Vitrella brassicaformis*	Eukaryota
UP000050934	*Limosilactobacillus secaliphilus*	Bacilli
UP000054361	fungal sp. No.11243*	Unidentified Fungi*
UP000054524	*Nematocida ausubeli*	Microsporidia
UP000054558	*Klebsormidium nitens*	Klebsormidiophyceae
UP000054937	*Pseudocohnilembus persalinus*	Oligohymenophorea
UP000063234	*Thermosulfidibacter takaii*	Thermosulfidibacteria
UP000070412	*Sarcoptes scabiei*	Arachnida
UP000070560	*Desulfofervidus auxilii**	Desulfofervidia*
UP000075320	*Bdellovibrio bacteriovorus*	Bdellovibrionia
UP000076078	*Tieghemostelium lacteum*	Eumycetozoa
UP000076632	*Xylona heveae*	Xylonomycetes
UP000076842	*Calocera cornea*	Dacrymycetes
UP000078348	*Blastocystis hominis*	Bigyra
UP000093080	*Dissulfuribacter thermophilus*	Dissulfuribacteria
UP000095255	*Desulfuribacillus stibiiarsenatis*	Desulfuribacillia
UP000095751	*Fragilariopsis cylindrus*	Bacillariophyceae
UP000182011	*Kryptonium thompsoni**	Kryptonia*
UP000185744	*Methanohalarchaeum thermophilum**	Methanonatronarchaeia
UP000185934	*Dehalogenimonas formicexedens*	Dehalococcoidia
UP000186303	*Malassezia sympodialis*	Malasseziomycetes
UP000190274	*Lachancea dasiensis*	Saccharomycetes
UP000192042	*Nitrospira japonica*	Nitrospiria
UP000192315	*Picrophilus oshimae*	Thermoplasmata
UP000193467	*Leucosporidium creatinivorum*	Microbotryomycetes
UP000198744	*Syntrophus gentianae*	Syntrophia
UP000199400	*Nannocystis exedens*	Myxococcota
UP000199452	*Williamwhitmania taraxaci*	Bacteroidia
UP000216329	*Bacteroidetes* bacterium B1*	Bacteroidota
UP000220158	*Plasmodium relictum*	Aconoidasida
UP000223071	*Tepidiforma thermophila*	Tepidiformia
UP000232323	*Chlamydomonas eustigma*	Chlorophyceae
UP000234329	*Acidithiobacillus marinus*	Acidithiobacillia
UP000237684	*Abditibacterium utsteinense*	Abditibacteriia
UP000238196	*Proteobacteria* bacterium 228*	Pseudomonadota
UP000240531	Archaeon SCG-AAA382B04*	Unidentified Archaea*
UP000245783	*Ceraceosorus guamensis*	Exobasidiomycetes
UP000246991	*Tuber magnatum*	Pezizomycetes
UP000248706	*Thermogemmatispora tikiterensis*	Ktedonobacteria
UP000250088	*Natrarchaeobaculum aegyptiacum*	Halobacteria
UP000251717	*Methanobrevibacter thaueri*	Methanobacteria
UP000253843	*Trichoplax* sp. H2*	Uniplacotomia
UP000256388	*Pelolinea submarina*	Anaerolineae
UP000262004	*Hydrogenophilus thermoluteolus*	Hydrogenophilia
UP000268059	*Intestinibaculum porci*	Erysipelotrichia
UP000270538	*Chryseobacterium* sp. 6424*	Flavobacteriia

Supplementary Table 2. (Continued.)

Identifier	Species name	Class name
UP000271624	*Dulcicalothrix desertica*	Cyanophyceae
UP000272943	*Aquitalea* sp. FJL05*	Betaproteobacteria
UP000276223	*Desulfosoma caldarium*	Syntrophobacteria
UP000287394	*Capsulimonas corticalis*	Armatimonadia
UP000289195	*Neochlamydia* sp. S13*	Chlamydiia
UP000294625	*Treponema* sp. J25*	Spirochaetia
UP000295063	*Anaerospora hongkongensis*	Negativicutes
UP000295281	*Actinorugispora endophytica*	Actinomycetes
UP000297299	*Botryotinia calthae*	Leotiomycetes
UP000298715	*Spiroplasma melliferum*	Mollicutes
UP000306585	*Mariprofundus erugo*	Zetaproteobacteria
UP000306912	*Culicoidibacter larvae*	Culicoidibacteria
UP000309626	*Bathymodiolus heckerae* thiotrophic gill symbiont*	Unidentified Bacteria*
UP000315010	*Planctomycetes* bacterium CA13*	Planctomycetota
UP000318571	*Tigriopus californicus*	Hexanauplia
UP000318582	*Powellomyces hirtus*	Chytridiomycetes
UP000322214	*Mariniblastus fucicola*	Planctomycetia
UP000322225	*Kwoniella shandongensis*	Tremellomycetes
UP000324789	*Acidobacteria* bacterium AB60*	Acidobacteriota
UP000334340	*Methylomirabilis lanthanidiphila**	Division NC10*
UP000355283	*Microchloropsis salina*	Eustigmatophyceae
UP000381693	*Methylacidimicrobium cyclopophantes*	Verrucomicrobiota
UP000412907	*Zixiibacteriota* bacterium*	Zixiibacteriota*
UP000424752	*Erwinia sorbitola*	Gammaproteobacteria
UP000448292	*Oceanidesulfovibrio indonesiensis*	Desulfovibrionia
UP000469346	*Dissulfurirhabdus thermomarina*	Deltaproteobacteria
UP000515312	*Alloacidobacterium dinghuense*	Terriglobia
UP000516360	*Dissulfurispira thermophila*	Thermodesulfovibrionia
UP000525652	*Puniceicoccus vermicola*	Opitutae
UP000528322	*Desulfurispira natronophila*	Chrysiogenetes
UP000563094	*Rufibacter quisquiliarum*	Cytophagia
UP000579846	*Sediminibacterium* sp. Gen4*	Chitinophagia
UP000582837	*Longimicrobium terrae*	Longimicrobiia
UP000591197	*Fusobacterium* sp. FSA-380-WT-3A*	Fusobacteriia
UP000594468	*Phototrophicus methaneseepsis*	Thermofonsia*
UP000596092	*Desulfobulbus oligotrophicus*	Desulfobulbia
UP000600918	*Vespula pensylvanica*	Insecta
UP000601171	*Paratissierella segnis*	Tissierellia
UP000603453	*Mucor saturninus*	Mucoromycetes
UP000610699	*Oligoflexia bacterium*	Oligoflexia
UP000627505	*Cyanobacteria* bacterium FACHB-502*	Cyanobacteriota
UP000644507	*Roseibacillus persicicus*	Verrucomicrobiae
UP000645828	*Nyctereutes procyonoides*	Mammalia
UP000654370	*Umbelopsis isabellina*	Umbelopsidomycetes
UP000663859	*Methylacidithermus pantelleriae**	Methylacidiphilae
UP000664203	*Alectoria fallacina*	Lecanoromycetes
UP000664859	*Tribonema minus*	Xanthophyceae
UP000678917	*Mycoplasmatota bacterium*	Mycoplasmatota
UP000726737	*Mortierella polycephala*	Mortierellomycetes
UP000756921	*Paraphaeosphaeria minitans*	Dothideomycetes
UP000789390	*Daphnia galeata*	Branchiopoda
UP000796880	*Rhamnella rubrinervis*	Magnoliopsida
UP000813600	*Phlebopus* sp. FC_14*	Agaricomycetes
UP000824219	*Hemibagrus wyckioides*	Actinopteri
UP000826003	*Gelria* sp. Kuro-4*	Clostridia
UP000827986	*Mauremys mutica*	Chordata
UP000886653	*Cronartium quercuum*	Pucciniomycetes
UP001151568	*Coemansia* sp. RSA 2050*	Kickxellomycetes
UP001152130	*Fusarium irregulare*	Sordariomycetes
UP000018888	*Rhizophagus irregularis*	Glomeromycetes
UP001152320	*Holothuria leucospilota*	Holothuroidea

*Candidate or unidentified classification

Supplementary Table 3. List of the 188 taxa and 974 sub-sampled sets of species defined from 9,163 quality-controlled proteomes. We provided corresponding NCBI taxonomy ID, rank and type (Bacteria or Fungi), along with the number of member species and sub-sampled sets.

ID	Scientific name	Rank	Type	Members	Sets
136	Spirochaetales	Order	Bacteria	35	1
237	*Flavobacterium*	Genus	Bacteria	73	2
265	*Paracoccus*	Genus	Bacteria	36	1
286	*Pseudomonas*	Genus	Bacteria	55	1
356	Hyphomicrobiales	Order	Bacteria	401	13
379	*Rhizobium*	Genus	Bacteria	34	1
433	Acetobacteraceae	Family	Bacteria	73	2
468	Moraxellaceae	Family	Bacteria	34	1
481	Neisseriaceae	Family	Bacteria	35	1
506	Alcaligenaceae	Family	Bacteria	50	1
543	Enterobacteriaceae	Family	Bacteria	41	1
641	Vibrionaceae	Family	Bacteria	48	1
662	*Vibrio*	Genus	Bacteria	35	1
815	Bacteroidaceae	Family	Bacteria	33	1
838	*Prevotella*	Genus	Bacteria	40	1
976	Bacteroidota	Phylum	Bacteria	894	29
1117	Cyanobacteriota	Phylum	Bacteria	178	5
1161	Nostocales	Order	Bacteria	44	1
1224	Pseudomonadota	Phylum	Bacteria	2733	91
1236	Gammaproteobacteria	Class	Bacteria	886	29
1239	Bacillota	Phylum	Bacteria	1493	49
1268	Micrococcaceae	Family	Bacteria	94	3
1300	Streptococcaceae	Family	Bacteria	52	1
1301	*Streptococcus*	Genus	Bacteria	40	1
1385	Bacillales	Order	Bacteria	559	18
1386	*Bacillus*	Genus	Bacteria	62	2
1485	*Clostridium*	Genus	Bacteria	105	3
1653	Corynebacteriaceae	Family	Bacteria	74	2
1663	*Arthrobacter*	Genus	Bacteria	40	1
1678	*Bifidobacterium*	Genus	Bacteria	43	1
1716	*Corynebacterium*	Genus	Bacteria	74	2
1760	Actinomycetes	Class	Bacteria	1762	58
1762	Mycobacteriaceae	Family	Bacteria	75	2
1763	*Mycobacterium*	Genus	Bacteria	40	1
1817	*Nocardia*	Genus	Bacteria	34	1
1839	*Nocardioides*	Genus	Bacteria	73	2
1873	*Micromonospora*	Genus	Bacteria	41	1
1883	*Streptomyces*	Genus	Bacteria	353	11
2004	Streptosporangiaceae	Family	Bacteria	59	1
2012	Thermomonosporaceae	Family	Bacteria	33	1
2037	Actinomycetales	Order	Bacteria	44	1
2049	Actinomycetaceae	Family	Bacteria	44	1
2062	Streptomycetaceae	Family	Bacteria	380	12
2070	Pseudonocardiaceae	Family	Bacteria	114	3
4890	Ascomycota	Phylum	Fungi	527	26
4891	Saccharomycetes	Class	Fungi	71	3
4892	Saccharomycetales	Order	Fungi	71	3
4893	Saccharomycetaceae	Family	Fungi	23	1
5042	Eurotiales	Order	Fungi	111	5
5052	*Aspergillus*	Genus	Fungi	59	2
5073	*Penicillium*	Genus	Fungi	42	2
5125	Hypocreales	Order	Fungi	104	5
5178	Helotiales	Order	Fungi	28	1
5204	Basidiomycota	Phylum	Fungi	153	7
5338	Agaricales	Order	Fungi	47	2
5455	*Colletotrichum*	Genus	Fungi	22	1
5506	*Fusarium*	Genus	Fungi	51	2
13687	*Sphingomonas*	Genus	Bacteria	85	2
28056	Micromonosporaceae	Family	Bacteria	104	3
28211	Alphaproteobacteria	Class	Bacteria	1302	43
28216	Betaproteobacteria	Class	Bacteria	534	17
28256	Halomonadaceae	Family	Bacteria	37	1
29547	Campylobacterota	Phylum	Bacteria	82	2

Supplementary Table 3. (Continued.)

ID	Scientific name	Rank	Type	Members	Sets
31953	Bifidobacteriaceae	Family	Bacteria	48	1
31957	Propionibacteriaceae	Family	Bacteria	40	1
31979	Clostridiaceae	Family	Bacteria	130	4
31989	Paracoccaceae	Family	Bacteria	200	6
32003	Nitrosomonadales	Order	Bacteria	61	2
32033	Xanthomonadaceae	Family	Bacteria	76	2
33882	*Microbacterium*	Genus	Bacteria	68	2
33958	Lactobacillaceae	Family	Bacteria	124	4
41297	Sphingomonadaceae	Family	Bacteria	191	6
44249	*Paenibacillus*	Genus	Bacteria	118	3
49546	Flavobacteriaceae	Family	Bacteria	320	10
57723	Acidobacteriota	Phylum	Bacteria	32	1
69277	Phyllobacteriaceae	Family	Bacteria	64	2
72274	Pseudomonadales	Order	Bacteria	85	2
72275	Alteromonadaceae	Family	Bacteria	34	1
72293	Helicobacteraceae	Family	Bacteria	30	1
74201	Verrucomicrobiota	Phylum	Bacteria	43	1
75682	Oxalobacteraceae	Family	Bacteria	66	2
76892	Caulobacteraceae	Family	Bacteria	36	1
80840	Burkholderiales	Order	Bacteria	372	12
80864	Comamonadaceae	Family	Bacteria	128	4
81852	Enterococcaceae	Family	Bacteria	46	1
82115	Rhizobiaceae	Family	Bacteria	67	2
84566	Sphingobacteriaceae	Family	Bacteria	110	3
84567	*Pedobacter*	Genus	Bacteria	34	1
84998	Coriobacteriia	Class	Bacteria	47	1
85004	Bifidobacteriales	Order	Bacteria	48	1
85006	Micrococcales	Order	Bacteria	474	15
85007	Mycobacteriales	Order	Bacteria	243	8
85008	Micromonosporales	Order	Bacteria	104	3
85009	Propionibacteriales	Order	Bacteria	147	4
85010	Pseudonocardiales	Order	Bacteria	114	3
85011	Kitasatosporales	Order	Bacteria	380	12
85012	Streptosporangiales	Order	Bacteria	115	3
85015	Nocardioidaceae	Family	Bacteria	96	3
85016	Cellulomonadaceae	Family	Bacteria	33	1
85021	Intrasporangiaceae	Family	Bacteria	33	1
85023	Microbacteriaceae	Family	Bacteria	220	7
85025	Nocardiaceae	Family	Bacteria	67	2
85030	Geodermatophilaceae	Family	Bacteria	31	1
91061	Bacilli	Class	Bacteria	825	27
91347	Enterobacterales	Order	Bacteria	106	3
92860	Pleosporales	Order	Fungi	46	2
110618	Nectriaceae	Family	Fungi	57	2
117743	Flavobacteriia	Class	Bacteria	373	12
117747	Sphingobacteriia	Class	Bacteria	110	3
119045	Methylobacteriaceae	Family	Bacteria	34	1
119060	Burkholderiaceae	Family	Bacteria	89	2
135613	Chromatiales	Order	Bacteria	71	2
135614	Xanthomonadales	Order	Bacteria	114	3
135619	Oceanospirillales	Order	Bacteria	110	3
135620	Oceanospirillaceae	Family	Bacteria	44	1
135621	Pseudomonadaceae	Family	Bacteria	71	2
135622	Alteromonadales	Order	Bacteria	118	3
135623	Vibrionales	Order	Bacteria	48	1
147541	Dothideomycetes	Class	Fungi	98	4
147545	Eurotiomycetes	Class	Fungi	143	7
147548	Leotiomycetes	Class	Fungi	32	1
147550	Sordariomycetes	Class	Fungi	164	8
155619	Agaricomycetes	Class	Fungi	97	4
165695	*Sphingobium*	Genus	Bacteria	30	1
165696	*Novosphingobium*	Genus	Bacteria	40	1
171549	Bacteroidales	Order	Bacteria	166	5
171552	Prevotellaceae	Family	Bacteria	64	2

Supplementary Table 3. (Continued.)

ID	Scientific name	Rank	Type	Members	Sets
186801	Clostridia	Class	Bacteria	557	18
186802	Eubacteriales	Order	Bacteria	321	10
186803	Lachnospiraceae	Family	Bacteria	131	4
186806	Eubacteriaceae	Family	Bacteria	33	1
186817	Bacillaceae	Family	Bacteria	293	9
186818	Planococcaceae	Family	Bacteria	42	1
186822	Paenibacillaceae	Family	Bacteria	159	5
186826	Lactobacillales	Order	Bacteria	266	8
194924	Desulfovibrionaceae	Family	Bacteria	32	1
200643	Bacteroidia	Class	Bacteria	189	6
200644	Flavobacteriales	Order	Bacteria	373	12
200666	Sphingobacteriales	Order	Bacteria	110	3
200940	Thermodesulfobacteriota	Phylum	Bacteria	117	3
201174	Actinomycetota	Phylum	Bacteria	1809	60
203682	Planctomycetota	Phylum	Bacteria	73	2
203683	Planctomycetia	Class	Bacteria	73	2
203691	Spirochaetota	Phylum	Bacteria	44	1
203692	Spirochaetia	Class	Bacteria	44	1
204432	Terriglobia	Class	Bacteria	31	1
204433	Terriglobales	Order	Bacteria	30	1
204441	Rhodospirillales	Order	Bacteria	143	4
204455	Rhodobacterales	Order	Bacteria	396	13
204457	Sphingomonadales	Order	Bacteria	274	9
204458	Caulobacterales	Order	Bacteria	36	1
206351	Neisseriales	Order	Bacteria	62	2
206389	Rhodocyclales	Order	Bacteria	37	1
213115	Desulfovibrionales	Order	Bacteria	39	1
213849	Campylobacterales	Order	Bacteria	78	2
216572	Oscillospiraceae	Family	Bacteria	84	2
335929	Erythrobacteraceae	Family	Bacteria	70	2
423349	*Mucilaginibacter*	Genus	Bacteria	36	1
563835	Chitinophagaceae	Family	Bacteria	68	2
681950	Glomerellaceae	Family	Fungi	22	1
768503	Cytophagia	Class	Bacteria	141	4
768507	Cytophagales	Order	Bacteria	141	4
909932	Negativicutes	Class	Bacteria	48	1
1028384	Glomerellales	Order	Fungi	22	1
1131492	Aspergillaceae	Family	Fungi	103	5
1643682	Geodermatophilales	Order	Bacteria	32	1
1706369	Cellvibrionales	Order	Bacteria	42	1
1737404	Tissierellia	Class	Bacteria	35	1
1737405	Tissierellales	Order	Bacteria	35	1
1775411	Rhodanobacteraceae	Family	Bacteria	38	1
1822464	*Paraburkholderia*	Genus	Bacteria	30	1
1853228	Chitinophagia	Class	Bacteria	68	2
1853229	Chitinophagales	Order	Bacteria	68	2
1853232	Hymenobacteraceae	Family	Bacteria	39	1
1866885	*Mycolicibacterium*	Genus	Bacteria	32	1
1890424	Synechococcales	Order	Bacteria	31	1
1913637	Mucoromycota	Phylum	Fungi	41	2
2691354	Pirellulales	Order	Bacteria	40	1
2726947	Mycosphaerellales	Order	Fungi	24	1
2762318	Weeksellaceae	Family	Bacteria	37	1
2854170	Roseobacteraceae	Family	Bacteria	196	6
2887326	Moraxellales	Order	Bacteria	34	1
2975441	Sphaerotilaceae	Family	Bacteria	39	1
3028117	Cyanophyceae	Class	Bacteria	178	5
3031449	Desulfovibrionia	Class	Bacteria	39	1
3031852	Epsilonproteobacteria	Class	Bacteria	80	2
3082720	Peptostreptococcales	Order	Bacteria	50	1
3085636	Lachnospirales	Order	Bacteria	138	4
				Total	
				30497	974

Supplementary Table 4. Detailed list of the 261 sets on which benchmark was performed. Link to the corresponding UniProt website is given for each proteome ID index.

Set No.	Taxon name	Proteome IDs
136.0	Spirochaetales	503, 811, 1807, 2967, 3571, 6546, 6852, 7254, 7383, 8212, 9222, 9223, 14541, 14605, 15620, 18680, 42527, 182360, 186400, 190395, 190423, 253869, 294625, 323824, 324209, 515827, 587760, 595917, 660053, 671908
237.1	*Flavobacterium*	5638, 6394, 30129, 30152, 31466, 93807, 184232, 184488, 198345, 198648, 216605, 237056, 237310, 244527, 244929, 244937, 245429, 245449, 247903, 271937, 289734, 296862, 297407, 316154, 557042, 609172, 625735, 630340, 675047, 1139260
265.0	*Paracoccus*	361, 15480, 32743, 183635, 183790, 191257, 198307, 199054, 199125, 199180, 199344, 199502, 218023, 233742, 234530, 234882, 252023, 253345, 283587, 285530, 293520, 309747, 316225, 321562, 434754, 442533, 478740, 556552, 608594, 640485
286.0	*Pseudomonas*	556, 686, 15503, 22611, 25241, 29493, 29499, 30063, 31652, 32531, 33462, 56648, 64137, 94259, 198407, 199524, 232228, 242957, 243063, 243359, 243778, 244064, 244119, 251762, 268166, 270661, 326483, 501989, 615613, 635983
356.12	Hyphomicrobiales	8952, 17669, 18524, 18542, 33187, 38011, 51058, 53043, 53675, 70354, 183447, 186364, 199236, 199466, 199667, 238563, 244780, 254764, 295097, 295238, 321085, 324738, 325303, 433101, 515290, 528286, 583454, 585507, 596427, 602124
379.0	*Rhizobium*	1936, 9319, 17669, 29602, 31368, 34908, 51333, 52057, 52066, 52122, 52161, 93137, 186894, 192550, 199253, 219167, 221890, 242816, 243756, 245252, 248925, 295383, 295799, 316801, 325303, 524535, 547048, 582090, 588925, 633219
433.0	Acetobacteraceae	245, 1176, 1963, 6375, 19250, 19760, 32668, 32679, 179145, 188604, 188879, 194822, 239724, 247565, 249065, 295023, 317078, 317214, 321405, 321746, 325255, 460715, 463253, 553193, 562254, 580654, 597459, 597507, 600101, 664073
468.0	Moraxellaceae	430, 546, 5740, 7477, 13117, 14568, 18415, 18418, 23785, 23788, 23795, 35860, 64939, 76238, 92616, 93391, 185895, 186001, 187495, 188357, 190435, 191094, 196536, 242317, 243900, 244223, 245977, 253940, 256774, 292423
481.0	Neisseriaceae	425, 535, 2768, 3009, 3019, 3344, 4088, 4105, 4207, 10457, 17813, 18554, 31390, 31392, 36027, 76625, 193118, 198238, 219669, 238938, 254209, 254293, 254651, 268229, 269923, 272771, 279284, 308891, 325536, 653156
506.0	Alcaligenaceae	1977, 16497, 19095, 19805, 29629, 36382, 76848, 78558, 93512, 184226, 194139, 196306, 214603, 216496, 216947, 235994, 236176, 238308, 244571, 283474, 288599, 294692, 439382, 494269, 502685, 559809, 580517, 608345, 675920, 739565
543.0	Enterobacteriaceae	260, 558, 625, 747, 1006, 1122, 1123, 1410, 2032, 2084, 2716, 5726, 6872, 7841, 8216, 10297, 14012, 27726, 28653, 29462, 29481, 36196, 37393, 53401, 187148, 280899, 302163, 516181, 654998, 659047
641.0	Vibrionaceae	537, 593, 2943, 16562, 29994, 31672, 32303, 33449, 33633, 35909, 36426, 37515, 51221, 78503, 92876, 94070, 94165, 94936, 184608, 193432, 198854, 215148, 219336, 232179, 235563, 235828, 326936, 464262, 694232, 1155586
662.0	*Vibrio*	584, 2493, 2943, 16562, 16567, 16895, 29994, 31672, 33673, 37515, 51221, 92876, 93173, 94070, 184608, 188276, 189475, 193432, 198854, 219336, 232179, 235563, 235828, 281112, 462621, 464262, 533102, 694232, 1139488, 1155586
815.0	Bacteroidaceae	1414, 2861, 3416, 6731, 7995, 14212, 17831, 17907, 17962, 18018, 18063, 18121, 18362, 18372, 18392, 18406, 18439, 56419, 92631, 95517, 95657, 184436, 195527, 196631, 262405, 264777, 279562, 284249, 324383, 651085
838.0	*Prevotella*	3327, 3829, 5697, 10862, 15929, 18046, 18054, 18062, 18111, 18116, 18147, 18183, 18184, 18343, 18353, 18357, 18375, 18727, 29597, 29732, 70533, 198560, 198779, 199373, 199625, 215645, 216632, 278983, 321612, 825483
976.24	Bacteroidota	588, 852, 3327, 7590, 8898, 17962, 18199, 32747, 75267, 76582, 187464, 198813, 199312, 199514, 236454, 241507, 244677, 249239, 249542, 253919, 255317, 263900, 266118, 270538, 286701, 295260, 297861, 317847, 437116, 503278
1117.3	Cyanobacteriota	268, 1423, 1566, 1961, 4344, 10475, 10478, 11180, 16960, 27395, 76925, 184315, 194922, 217698, 218238, 226442, 238762, 239001, 239589, 276103, 318453, 594557, 607397, 620559, 624057, 639877, 640021, 641405, 707356, 729701
1161.0	Nostocales	1191, 1511, 2483, 10378, 10380, 10390, 10474, 10475, 10477, 53372, 65745, 76925, 198313, 218238, 218621, 218785, 239589, 245124, 252906, 268857, 271624, 276103, 282767, 593846, 596768, 599391, 629098, 641405, 683511, 729701
1224.73	Pseudomonadota	1695, 1844, 2745, 5740, 11834, 14568, 19113, 31843, 35397, 58074, 180009, 183107, 195667, 198645, 199054, 218896, 241074, 244817, 245708, 283474, 284395, 285530, 310016, 321172, 465810, 543279, 546031, 561045, 606196, 631034
1236.27	Gammaproteobacteria	1122, 1947, 6286, 10320, 32414, 35860, 54457, 55136, 59419, 61457, 70250, 92671, 181743, 198575, 198706, 218896, 234640, 235547, 242642, 253740, 254575, 281112, 282106, 302163, 310227, 316365, 321272, 501602, 596063, 635983
1239.36	Bacillota	3195, 3330, 4069, 6316, 7523, 11124, 11912, 17747, 18136, 18364, 30408, 30832, 32409, 37233, 51230, 51657, 51813, 78534, 95380, 195141, 199476, 217785, 288024, 323257, 502248, 562464, 595468, 656813, 679779, 681343

Supplementary Table 4. (Continued.)

Set No.	Taxon name	Proteome IDs
1268.2	Micrococcaceae	30301, 30982, 53171, 60433, 95776, 177882, 190514, 192359, 219947, 247286, 277032, 280861, 283134, 292685, 295411, 305233, 307000, 325957, 326852, 460157, 516404, 516421, 544090, 600171, 633136, 638848, 655366, 664164, 676885, 1139502
1300.0	Streptococcaceae	585, 586, 750, 2196, 2814, 2815, 3330, 7946, 8520, 9077, 70387, 70638, 72050, 182508, 186437, 198604, 218181, 218689, 242246, 254082, 254634, 254924, 269148, 269374, 318879, 439550, 522720, 562464, 563349, 644875
1301.0	*Streptococcus*	449, 585, 586, 750, 1170, 1452, 2148, 2814, 2815, 4514, 5388, 7946, 70387, 70638, 72050, 182508, 186437, 198604, 249495, 254082, 254634, 254924, 269148, 279194, 281771, 291494, 522720, 563349, 644875, 660801
1385.16	Bacillales	3445, 6315, 8816, 17973, 18296, 27985, 28863, 35996, 78534, 92623, 180019, 180199, 189761, 198734, 198936, 215059, 248066, 258625, 265711, 270468, 275473, 282076, 295689, 437773, 515679, 593626, 618460, 676456, 829401, 1084197
1386.1	*Bacillus*	606, 27602, 27822, 36953, 60713, 67625, 95209, 180019, 180199, 185615, 199133, 199578, 216230, 219546, 226441, 253314, 258625, 263943, 265711, 269701, 325032, 437773, 472971, 481043, 531594, 575961, 666543, 678477, 678491, 679638
1485.2	*Clostridium*	818, 1986, 2730, 8937, 10420, 17118, 17809, 17939, 18022, 18023, 18136, 18268, 19426, 30009, 75531, 76603, 175744, 184869, 192468, 220840, 254664, 260680, 286268, 287890, 306888, 320543, 545014, 559354, 580568, 594441
1653.1	Corynebacteriaceae	545, 582, 1473, 2198, 4208, 6078, 10445, 14809, 15388, 16943, 17152, 23703, 29914, 35199, 70591, 76947, 177657, 182237, 185479, 217209, 238368, 244989, 278422, 320791, 424462, 516320, 594681, 645966, 658613, 762474
1663.0	*Arthrobacter*	30301, 53253, 54460, 176251, 181917, 190514, 195913, 199258, 237061, 239972, 244710, 247980, 273807, 280861, 283134, 295411, 295511, 296209, 305124, 306826, 317144, 326852, 543556, 544090, 547458, 664164, 676885, 683653, 687525, 692783
1678.0	*Bifidobacterium*	439, 3191, 8702, 28730, 29004, 29014, 29033, 29046, 29050, 29052, 29060, 29067, 29096, 29108, 30636, 216352, 216444, 216451, 229095, 229239, 242610, 252530, 287470, 287533, 287609, 288052, 326336, 441772, 482084, 718821
1716.0	*Corynebacterium*	492, 4218, 10445, 14809, 15388, 17152, 19222, 33457, 35368, 50488, 60016, 70591, 76947, 177600, 177657, 199350, 221653, 244989, 247696, 251577, 254467, 278422, 312032, 317518, 326711, 439070, 515743, 516320, 645966, 762474
1760.24	Actinomycetes	5063, 7076, 10411, 16033, 23703, 31501, 50821, 51005, 51073, 94243, 175826, 184452, 185596, 237697, 254869, 262621, 281230, 292235, 319746, 321701, 323454, 425099, 431080, 473525, 537260, 541033, 581206, 606172, 653493, 694501
1762.0	Mycobacteriaceae	757, 1020, 1419, 1584, 9224, 36176, 36334, 51677, 70146, 70612, 77143, 94224, 192566, 192801, 193090, 193564, 220914, 268285, 279306, 282551, 295165, 320513, 323700, 430146, 466445, 466517, 467006, 467105, 467130, 1064782
1763.0	*Mycobacterium*	1020, 1190, 1419, 1584, 1988, 25947, 36334, 51677, 53444, 70146, 77143, 92025, 92027, 92060, 92151, 93629, 94224, 94976, 179734, 192566, 193990, 194000, 223528, 320513, 465361, 466396, 467105, 467130, 554965, 682202
1817.0	*Nocardia*	6304, 6820, 17048, 19150, 19401, 76512, 188836, 215506, 219565, 246410, 252586, 254869, 255355, 255467, 266677, 279275, 294615, 429627, 431401, 432464, 438448, 503540, 515512, 540412, 540698, 565715, 586827, 612956, 638263, 655751
1839.0	*Nocardioides*	15971, 33772, 50886, 51395, 51495, 51699, 77868, 198649, 198832, 199004, 246018, 279994, 283644, 291189, 294853, 304990, 307087, 308550, 324351, 325003, 326325, 433406, 473325, 502035, 530424, 535511, 537326, 549911, 616839, 640489
1873.0	*Micromonospora*	1908, 3448, 10307, 32254, 70620, 186004, 186700, 198210, 198217, 198251, 198765, 198937, 199001, 199393, 199408, 199413, 199699, 240342, 242415, 258607, 262621, 277671, 281230, 281726, 283832, 285744, 295555, 427831, 478148, 578819
1883.4	*Streptomyces*	2357, 4217, 9036, 10411, 14390, 14410, 52945, 53429, 78145, 94479, 186200, 189065, 189443, 199155, 217446, 218662, 235945, 265325, 281963, 294981, 326553, 472335, 481583, 501663, 637788, 645555, 656732, 677732, 689511, 693098
2004.0	Streptosporangiaceae	6640, 18869, 77701, 190797, 198923, 198953, 199361, 236732, 249304, 253094, 253303, 284824, 286861, 295157, 306628, 308705, 530928, 540685, 555564, 562352, 568380, 578449, 586042, 605992, 610966, 640052, 645217, 655287, 674234, 1143474
2012.0	Thermomonosporaceae	183413, 198318, 236723, 242367, 251891, 256661, 261811, 262882, 272400, 282674, 288610, 294513, 295578, 295596, 295623, 316096, 316706, 323380, 432015, 468735, 487268, 501240, 539313, 546324, 572680, 579250, 614047, 669179, 1165074, 1165124
2037.0	Actinomycetales	3045, 3994, 5351, 6742, 9888, 10301, 13015, 18843, 54404, 78368, 176288, 182744, 185628, 186785, 245283, 250192, 266895, 270021, 271272, 273303, 276899, 280344, 284680, 293036, 594637, 595895, 595973, 614239, 627538, 683503
2049.0	Actinomycetaceae	2941, 3045, 3822, 5351, 6742, 9888, 10301, 16495, 54404, 78368, 176288, 176960, 184291, 185612, 185628, 186785, 250192, 266895, 269542, 271272, 273303, 276899, 280444, 284680, 293036, 594637, 595973, 614239, 627538, 683503

Supplementary Table 4. (Continued.)

Set No.	Taxon name	Proteome IDs
2062.2	Streptomycetaceae	4184, 8043, 14629, 28341, 37702, 37982, 51136, 54011, 217789, 237426, 240682, 245455, 250268, 271004, 276963, 295370, 317940, 435531, 450869, 472335, 473262, 481552, 509480, 509509, 528419, 600365, 603708, 608024, 630936, 655443
2070.0	Pseudonocardiaceae	328, 5087, 6281, 7809, 13968, 19277, 31419, 33409, 62973, 184363, 185596, 186040, 192591, 194360, 198348, 198582, 198967, 199494, 199614, 204221, 238362, 239494, 249915, 274515, 295444, 325787, 478898, 550714, 650035, 660680
4890.0	Ascomycota	36893, 55045, 184073, 186583, 190274, 231358, 240493, 241462, 248349, 248423, 266453, 544331, 582016, 738349, 774326, 788993, 800041, 813383, 1140510, 1152130
4890.1	Ascomycota	6701, 16922, 30104, 182235, 191612, 244722, 256690, 298493, 315783, 326289, 326340, 567885, 569620, 631181, 664169, 664534, 756132, 799441, 811619, 1140502
4890.10	Ascomycota	8062, 8181, 8513, 16923, 27730, 30663, 37505, 184546, 222788, 244309, 246702, 249402, 266272, 452235, 541154, 558688, 694255, 800092, 813426, 813461
4890.11	Ascomycota	1197, 16801, 19471, 28524, 30706, 53411, 54466, 95085, 295703, 297299, 305883, 310108, 319663, 326198, 554235, 654913, 676310, 736672, 799772, 829364
4890.12	Ascomycota	1067, 9058, 54771, 223968, 237438, 241587, 241690, 243732, 284375, 288168, 325902, 326931, 327118, 434172, 515153, 748025, 782241, 800039, 1147733, 1147747
4890.13	Ascomycota	2762, 7115, 7703, 16932, 19375, 20467, 31176, 53328, 76632, 77069, 91967, 243015, 249363, 249829, 325579, 758155, 800038, 809789, 1147760, 1149163
4890.14	Ascomycota	709, 8066, 11761, 15100, 16935, 34112, 50424, 70168, 190831, 191342, 297527, 326757, 398389, 578531, 737391, 758603, 799539, 800040, 1056384, 1146351
4890.15	Ascomycota	314, 598, 1471, 5627, 9096, 19484, 54821, 76881, 94455, 218381, 234585, 285146, 593570, 664203, 664521, 697127, 777438, 799424, 799537, 1152049
4890.16	Ascomycota	2037, 53617, 76837, 78340, 78544, 95728, 191408, 191691, 193144, 193689, 194361, 237481, 249293, 325492, 481858, 694050, 701801, 813444, 1149165, 1150942
4890.17	Ascomycota	689, 1610, 1631, 1881, 1997, 5220, 6702, 37136, 37696, 54302, 76874, 189513, 192386, 219602, 235786, 325672, 509704, 700596, 749293, 785799
4890.18	Ascomycota	1640, 2499, 8784, 9097, 54544, 70121, 76552, 184300, 191518, 224080, 226031, 265631, 266234, 285775, 286921, 326924, 613401, 696573, 722485, 1056012
4890.19	Ascomycota	560, 1745, 2311, 5222, 7796, 9882, 24533, 94112, 177622, 237631, 243797, 248340, 250266, 596902, 637239, 696280, 720189, 799777, 1150879, 1150941
4890.2	Ascomycota	267, 1261, 2258, 2866, 8536, 19487, 37904, 70720, 78343, 178129, 190312, 241107, 242519, 253071, 275385, 319160, 510647, 606974, 799291, 799423
4890.20	Ascomycota	6310, 6911, 7304, 8142, 15530, 16934, 19478, 54516, 70133, 214365, 225277, 233524, 235728, 245910, 246991, 247810, 248961, 504637, 531561, 800036
4890.21	Ascomycota	591, 2038, 2530, 27920, 42958, 77535, 94389, 184383, 241818, 247233, 253472, 509510, 562929, 572817, 604273, 730381, 799429, 799437, 827724, 1147746
4890.22	Ascomycota	599, 2036, 7322, 19804, 54304, 70501, 76584, 183809, 192596, 230249, 236621, 316270, 672032, 730481, 790833, 799324, 799436, 799438, 799771, 1153618
4890.23	Ascomycota	2428, 5426, 16936, 18001, 30651, 94801, 184304, 215289, 235371, 238813, 266152, 275078, 277212, 292447, 308549, 325780, 624404, 769157, 769528, 824596
4890.24	Ascomycota	1805, 7978, 18087, 53342, 92555, 95038, 193240, 235672, 238350, 252748, 253729, 286045, 286134, 290900, 622797, 635477, 654918, 756346, 1148299, 1152087
4890.25	Ascomycota	1798, 1996, 6753, 16928, 30106, 30143, 53029, 70700, 234275, 287544, 297452, 309340, 326532, 605986, 626568, 641853, 781932, 830671, 1140562, 1147752
4890.3	Ascomycota	5206, 5666, 6790, 8984, 16800, 19376, 30641, 34291, 54342, 242791, 326565, 326799, 431533, 547976, 750502, 756921, 813427, 1149074, 1149079, 1152649
4890.4	Ascomycota	724, 5221, 6039, 8782, 11096, 14480, 53259, 76744, 78397, 94526, 94569, 184188, 240883, 243723, 574317, 699042, 799770, 837801, 1152300, 1152885
4890.5	Ascomycota	2035, 2668, 8673, 30672, 54383, 182334, 184356, 191024, 191285, 226192, 241546, 242955, 276215, 283383, 297280, 310066, 660729, 701341, 717696, 1154252
4890.6	Ascomycota	15441, 16931, 27002, 77248, 184330, 188318, 236664, 243498, 254866, 283841, 283895, 326950, 532311, 653565, 661057, 714618, 799757, 799764, 1141434, 1147782
4890.7	Ascomycota	559, 707, 2497, 16933, 30753, 30816, 36947, 53841, 75602, 78576, 184499, 194280, 256645, 276864, 277580, 297229, 326268, 504636, 630445, 800035
4890.8	Ascomycota	2669, 19473, 33647, 94444, 179179, 182658, 186955, 191672, 267821, 283090, 287144, 297716, 307173, 546213, 799750, 799767, 800082, 800094, 800235, 1149954
4890.9	Ascomycota	1294, 6706, 9328, 27238, 53599, 94285, 176998, 196158, 215305, 234474, 517252, 536711, 557566, 593566, 639643, 651452, 799439, 800096, 801428, 1150904
4891.0	Saccharomycetes	559, 1997, 2311, 2866, 7703, 94112, 94285, 94801, 95085, 95728, 189513, 190274, 190831, 244309, 290900, 398389, 510647, 662931, 769528, 837801
4891.1	Saccharomycetes	591, 599, 707, 709, 1640, 1996, 5221, 5222, 19375, 92555, 94389, 95038, 238350, 249293, 253472, 307173, 697127, 769157, 790833, 1152885
4891.2	Saccharomycetes	598, 1300, 2036, 2037, 2428, 5220, 5627, 5666, 6310, 9328, 94455, 182334, 191024, 196158, 230249, 285775, 286412, 292447, 509704, 774326

Supplementary Table 4. (Continued.)

Set No.	Taxon name	Proteome IDs
4892.0	Saccharomycetales	689, 707, 2037, 5220, 6310, 8673, 9328, 19375, 94112, 94285, 94801, 189513, 196158, 249293, 253472, 398389, 509704, 662931, 694255, 774326
4892.1	Saccharomycetales	314, 591, 599, 1300, 1997, 2311, 5221, 5222, 5627, 8536, 92555, 95085, 182334, 190274, 285775, 286412, 292447, 769157, 769528, 837801
4892.2	Saccharomycetales	559, 709, 1640, 2036, 2258, 2428, 5666, 6790, 7703, 54304, 94389, 94455, 95038, 191024, 230249, 244309, 307173, 510647, 697127, 788993
4893.0	Saccharomycetaceae	267, 591, 689, 1640, 2036, 2311, 2428, 2866, 5220, 5627, 5666, 6310, 8536, 19375, 54304, 190274, 190831, 196158, 509704, 510647
5042.0	Eurotiales	9882, 18001, 30143, 37505, 179179, 184073, 184383, 184499, 186955, 190312, 191691, 249402, 325579, 630445, 637239, 701341, 1147746, 1147752, 1150879, 1150942
5042.1	Eurotiales	1294, 1745, 55045, 184188, 184304, 184546, 191342, 191408, 191672, 247810, 249829, 319663, 326289, 326532, 326950, 509510, 641853, 653565, 1147782, 1149074
5042.2	Eurotiales	6702, 36893, 70168, 94569, 191518, 215289, 234275, 234585, 248961, 249363, 256690, 283841, 286921, 325672, 326565, 631181, 1148299, 1149165, 1150941, 1153618
5042.3	Eurotiales	560, 6706, 19376, 37696, 42958, 214365, 215305, 231358, 246702, 248340, 248423, 253729, 325780, 326931, 541154, 1146351, 1149079, 1149954, 1150904, 1154252
5042.4	Eurotiales	724, 2530, 6701, 19804, 30104, 34291, 54383, 177622, 184300, 188318, 191612, 218381, 234474, 247233, 326268, 326799, 327118, 1147733, 1147760, 1149163
5052.0	*Aspergillus*	6701, 19804, 36893, 37505, 94569, 184499, 184546, 188318, 215289, 231358, 234275, 234474, 246702, 249402, 256690, 286921, 326532, 452235, 630445, 637239
5052.1	*Aspergillus*	6706, 34291, 54771, 184300, 184304, 184356, 215305, 248340, 248423, 248961, 253729, 325579, 325672, 325780, 326799, 326931, 327118, 541154, 641853, 661057
5073.0	*Penicillium*	9882, 30143, 42958, 55045, 186955, 191285, 191612, 191672, 218381, 631181, 701341, 1147733, 1147746, 1147747, 1147760, 1147782, 1149163, 1149954, 1150879, 1153618
5073.1	*Penicillium*	724, 19376, 30104, 37696, 70168, 177622, 191408, 191518, 191691, 1141434, 1146351, 1147752, 1148299, 1149074, 1149079, 1150904, 1150941, 1150942, 1152649, 1154252
5125.0	Hypocreales	5206, 7115, 7978, 16801, 27002, 28524, 36947, 78397, 78544, 236664, 532311, 557566, 562929, 567885, 569620, 730481, 737391, 813383, 813427, 824596
5125.1	Hypocreales	30106, 54544, 76744, 219602, 240493, 252748, 265631, 287544, 582016, 622797, 626568, 635477, 694050, 696573, 717696, 748025, 750502, 782241, 813444, 1152130
5125.2	Hypocreales	1610, 2499, 8984, 30663, 31176, 50424, 70720, 76874, 236621, 243498, 245910, 266272, 287144, 574317, 593570, 720189, 722485, 736672, 1140502, 1152087
5125.3	Hypocreales	9097, 16800, 34112, 37904, 76580, 76881, 78340, 226192, 241546, 241587, 253071, 277212, 536711, 544331, 554235, 558688, 604273, 738349, 777438, 829364
5125.4	Hypocreales	2762, 5426, 9096, 16928, 30753, 37136, 91967, 235728, 237481, 241690, 266152, 266234, 315783, 517252, 547976, 605986, 749293, 811619, 827724, 1152049
5178.0	Helotiales	1798, 178129, 184330, 235371, 235672, 235786, 238813, 241818, 242519, 254866, 256645, 297280, 297299, 297452, 326757, 531561, 624404, 696280, 701801, 1152300
5204.0	Basidiomycota	561, 2149, 53477, 54097, 62823, 76761, 76871, 78595, 184267, 198372, 245768, 249464, 256964, 284706, 294933, 305067, 307440, 521943, 629468, 1063166
5204.1	Basidiomycota	1072, 1861, 6352, 14071, 53257, 53890, 53989, 77086, 193218, 194127, 199069, 218811, 235388, 242287, 250043, 279236, 287166, 308652, 313359, 759537
5204.2	Basidiomycota	5242, 17559, 53392, 53424, 53558, 53611, 53819, 54166, 54477, 92583, 92666, 193986, 217199, 305948, 324022, 623687, 664032, 714275, 807025, 1142393
5204.3	Basidiomycota	17200, 30669, 54549, 76154, 76842, 77521, 94020, 218334, 219338, 310668, 324748, 567179, 609673, 703269, 772434, 807353, 807769, 813600, 815604, 886653
5204.4	Basidiomycota	8493, 16930, 30653, 53712, 54270, 54485, 77684, 92730, 186303, 237144, 245783, 246740, 298030, 323386, 365756, 559027, 736335, 765509, 812287, 813824
5204.5	Basidiomycota	7431, 9131, 15241, 27361, 54564, 76727, 76738, 94043, 94065, 183567, 193067, 217790, 249723, 284842, 322225, 322482, 719766, 724874, 807469, 1049176
5204.6	Basidiomycota	27073, 27195, 27222, 27265, 30671, 54007, 54018, 54538, 54988, 76532, 78113, 245771, 311382, 320762, 521872, 799118, 807306, 807342, 823399, 1150266
5338.0	Agaricales	27073, 27222, 53424, 54549, 76154, 242287, 283269, 284706, 284842, 298030, 307440, 567179, 629468, 724874, 807025, 807342, 807469, 812287, 1049176, 1150266
5338.1	Agaricales	1861, 8493, 53712, 54007, 54270, 54477, 54988, 218334, 305067, 308652, 320762, 521872, 521943, 623687, 664032, 772434, 807353, 813824, 1063166, 1142393
5455.0	*Colletotrichum*	8782, 11096, 14480, 15530, 20467, 27238, 70121, 76552, 76584, 186583, 295703, 305883, 326340, 434172, 613401, 639643, 654918, 699042, 781932, 830671
5506.0	*Fusarium*	9096, 9097, 16800, 30753, 37904, 91967, 219602, 241587, 266152, 277212, 536711, 546213, 547976, 569620, 605986, 622797, 736672, 737391, 813427, 1152087
5506.1	*Fusarium*	5206, 30663, 70720, 236664, 245910, 265631, 266234, 287144, 288168, 532311, 544331, 558688, 574317, 582016, 593570, 604273, 635477, 730481, 750502, 1140502

Supplementary Table 4. (Continued.)

Set No.	Taxon name	Proteome IDs
13687.1	*Sphingomonas*	33200, 50871, 51371, 51455, 51642, 51854, 51880, 185161, 188729, 194475, 198281, 198824, 198912, 199586, 218151, 218323, 241167, 244162, 266693, 286100, 286883, 315673, 321250, 436053, 515367, 529795, 558192, 613564, 623067, 1139410
28056.2	Micromonosporaceae	235, 3448, 5440, 53244, 186004, 197174, 198251, 198362, 199408, 199699, 242415, 287606, 295555, 482800, 542742, 578112, 578819, 587527, 598146, 619260, 623608, 629619, 635606, 649753, 660611, 676360, 680286, 680866, 1143480, 1165079
28211.24	Alphaproteobacteria	1302, 1591, 5713, 8850, 17808, 28638, 35287, 51777, 53043, 53455, 184221, 198703, 198912, 231259, 237344, 244932, 254889, 284322, 317894, 320653, 471755, 530268, 533469, 538147, 546701, 547048, 551327, 554286, 609121, 1144323
28216.16	Betaproteobacteria	7883, 8392, 51759, 63254, 77549, 184226, 186110, 197446, 197468, 198781, 199646, 229897, 237839, 239406, 241193, 247811, 266302, 288813, 295525, 297839, 301751, 305539, 318422, 325536, 444316, 487350, 515986, 559809, 664731, 681964
28256.0	Halomonadaceae	4512, 14463, 19113, 63387, 77875, 179615, 184346, 187327, 190911, 198500, 198641, 198654, 198693, 199040, 199677, 218896, 219993, 235346, 267342, 276016, 287023, 321272, 321275, 327197, 437638, 448235, 487929, 501340, 518892, 595089
29547.1	Campylobacterota	422, 760, 2714, 5085, 7032, 7727, 8139, 15520, 18143, 29714, 92701, 92884, 194141, 194309, 199227, 216568, 217944, 229442, 230780, 233248, 240535, 251062, 253850, 255139, 256695, 256842, 290191, 502820, 503264, 593836
31953.0	Bifidobacteriaceae	439, 2456, 5777, 6173, 8693, 16519, 29004, 29033, 29046, 29050, 29052, 29055, 29067, 29080, 216352, 216444, 216451, 216454, 229095, 229239, 231451, 242610, 243657, 252530, 287533, 287609, 326336, 441772, 469292, 482084
31957.0	Propionibacteriaceae	936, 7947, 14417, 93501, 184512, 187142, 188235, 188324, 188342, 199475, 215896, 216300, 216311, 252770, 273044, 274803, 275256, 277858, 291933, 292373, 295371, 316196, 317638, 319263, 425099, 435304, 523079, 527616, 569914, 613840
31979.3	Clostridiaceae	1325, 3081, 8937, 17118, 17991, 18047, 18076, 18120, 30009, 50326, 52015, 76603, 77407, 175744, 184035, 184526, 190080, 190748, 198524, 198597, 199127, 239471, 286268, 286369, 304999, 306888, 308489, 324646, 664218, 724672
31989.2	Paracoccaceae	4507, 30004, 50471, 183002, 186098, 194664, 198417, 199125, 201838, 207598, 215377, 215760, 230388, 234539, 237655, 244810, 244932, 245680, 279673, 282002, 293520, 316225, 442533, 478183, 484076, 572377, 598196, 619033, 619079, 638981
32003.1	Nitrosomonadales	383, 1235, 1629, 2440, 2718, 2743, 5019, 12179, 31637, 56322, 70578, 77545, 77549, 185062, 195558, 198620, 198729, 198764, 198772, 198814, 199561, 199646, 245081, 248024, 275137, 295135, 316473, 316827, 501534, 681964
32033.1	Xanthomonadaceae	1890, 5870, 8632, 29385, 30017, 30518, 33067, 50805, 51386, 51738, 51802, 61569, 218968, 219374, 239898, 249447, 251842, 262917, 269708, 289784, 291286, 291562, 315167, 316584, 317199, 319980, 322165, 519004, 639274, 680299
33882.0	*Microbacterium*	24001, 28883, 33900, 34098, 51381, 93355, 187047, 196320, 199183, 199422, 215463, 245623, 252505, 253508, 273158, 297593, 298650, 318564, 320235, 321196, 321225, 325827, 326838, 422989, 432812, 440939, 526083, 537775, 633205, 657592
33958.2	Lactobacillaceae	432, 1652, 1991, 4069, 4115, 4567, 5444, 17248, 18559, 37778, 50911, 50969, 51412, 51461, 51638, 51639, 51672, 51697, 51733, 51845, 51931, 51992, 52012, 52013, 94714, 199376, 290602, 294321, 295257, 1139006
41297.5	Sphingomonadaceae	4728, 11816, 13072, 37930, 77614, 188729, 194475, 218366, 238954, 253918, 266860, 276254, 282837, 286100, 286883, 287772, 298213, 319092, 326488, 426325, 509198, 546701, 552700, 552757, 554342, 570166, 571950, 575068, 578569, 676996
44249.2	*Paenibacillus*	3445, 3900, 7523, 28123, 29409, 29453, 29512, 32320, 36611, 37688, 76927, 92024, 92663, 93309, 187480, 190188, 199177, 215509, 244407, 252415, 279446, 293568, 295418, 307943, 325218, 450917, 490800, 502136, 608782, 676098
49546.0	Flavobacteriaceae	3023, 3053, 3730, 5938, 8297, 32229, 45051, 93510, 93807, 94750, 182510, 184516, 195470, 198521, 198999, 219193, 219559, 244937, 245429, 248054, 256629, 276603, 290889, 295479, 321734, 321790, 535020, 761667, 1139260, 1143545
57723.0	Acidobacteriota	343, 2207, 2432, 6056, 6844, 7113, 31518, 182427, 198356, 199024, 236728, 253606, 269669, 279598, 289437, 290253, 292958, 295210, 321820, 513480, 515312, 515460, 515496, 540989, 568106, 589520, 593892, 647241, 648801, 1059380
69277.1	Phyllobacteriaceae	3250, 4291, 6786, 50665, 50670, 54487, 70107, 94412, 179771, 182840, 191905, 199768, 215931, 218091, 235507, 238563, 242763, 244404, 278398, 281647, 293719, 295131, 312852, 317542, 323300, 595886, 630142, 636264, 643405, 1149009
72274.1	Pseudomonadales	233, 556, 686, 2515, 15503, 16560, 30980, 32239, 33462, 63953, 64137, 68210, 94259, 184497, 185766, 198407, 198706, 199290, 231409, 232228, 243359, 244119, 270661, 278332, 280615, 315235, 327522, 463138, 476718, 615613
72275.0	Alteromonadaceae	6263, 6327, 9282, 11864, 19276, 35062, 37600, 53586, 56090, 68447, 70299, 175691, 176037, 184520, 219285, 233828, 244441, 245728, 247426, 256561, 275281, 291390, 293649, 298049, 317204, 601768, 606935, 631300, 664654, 664717

Supplementary Table 4. (Continued.)

Set No.	Taxon name	Proteome IDs
72293.0	Helicobacteraceae	422, 429, 1522, 2495, 5085, 7032, 7934, 8387, 18143, 18688, 18731, 29707, 29714, 29920, 92701, 92884, 216091, 216568, 218502, 249746, 251062, 251881, 255139, 256379, 256421, 256695, 256765, 256842, 257067, 319322
74201.0	Verrucomicrobiota	1031, 4837, 7013, 71392, 78486, 95228, 199432, 217265, 239907, 244023, 306196, 315648, 321577, 475117, 478417, 500777, 501812, 525652, 526501, 546464, 557872, 590740, 600139, 603141, 617628, 642829, 644507, 658278, 676169, 825051
75682.0	Oxalobacteraceae	329, 5089, 27604, 28843, 31572, 34315, 51728, 51760, 56897, 190838, 197535, 198284, 199391, 214937, 228593, 240505, 241421, 244112, 265955, 274350, 285190, 294829, 297258, 298438, 444318, 451565, 484015, 502415, 534388, 622890
76892.0	Caulobacteraceae	1816, 1868, 2696, 17808, 17812, 17826, 17837, 50923, 51150, 51182, 51604, 56905, 77603, 195546, 228945, 247763, 249254, 249524, 249725, 249842, 317223, 320100, 431269, 482044, 545037, 548978, 566663, 622580, 662572, 663918
80840.1	Burkholderiales	5045, 16497, 27020, 27834, 29629, 50580, 51403, 51759, 51826, 72741, 214603, 216607, 243193, 252174, 274884, 291078, 298438, 439382, 444316, 502415, 521868, 529637, 542125, 574067, 580517, 588051, 623397, 627205, 675920, 739565
80864.0	Comamonadaceae	374, 2482, 3856, 4811, 7938, 8332, 51275, 51403, 51912, 53300, 70169, 72741, 94760, 185680, 190750, 194358, 194432, 198552, 199369, 199766, 216607, 239709, 297564, 316798, 464549, 501978, 530032, 541185, 623043, 664731
81852.0	Enterococcaceae	1415, 8456, 10296, 12675, 13782, 14127, 15961, 17415, 18126, 95256, 181884, 182077, 182149, 188246, 195141, 195918, 196151, 236214, 286773, 287101, 287857, 288028, 288197, 290567, 298615, 321556, 500890, 622610, 637757, 674938
82115.0	Rhizobiaceae	813, 1054, 1596, 1936, 1976, 9319, 10792, 17669, 28181, 29602, 31368, 34908, 51333, 52122, 52161, 52167, 186894, 241247, 248925, 264280, 295238, 295383, 316801, 439983, 547048, 582090, 588925, 663351, 663401, 680848
84566.0	Sphingobacteriaceae	310, 3664, 36014, 50543, 186720, 198670, 198785, 199072, 199226, 199666, 238034, 248198, 253209, 268007, 284120, 286701, 291117, 293331, 293347, 307244, 308196, 310477, 323829, 462014, 505355, 515877, 538158, 601292, 619078, 662074
84567.0	*Pedobacter*	852, 3664, 28007, 36014, 50543, 71561, 78459, 94313, 192756, 198039, 198850, 199572, 199666, 240912, 244301, 245379, 248198, 284120, 291117, 293347, 295499, 295620, 295668, 307244, 308181, 309488, 310477, 601055, 601292, 651668
84998.0	Coriobacteriia	333, 954, 960, 2026, 3446, 3560, 4830, 6069, 6851, 12651, 14204, 16638, 18266, 31121, 54078, 70675, 182975, 190585, 195399, 195781, 195889, 253805, 269591, 273154, 321063, 400317, 468668, 486032, 503297, 681649
85004.0	Bifidobacteriales	439, 2456, 3191, 5777, 6173, 8693, 8702, 28730, 29014, 29033, 29080, 29082, 29093, 29108, 30636, 216352, 216444, 216451, 228755, 229095, 231451, 240228, 242610, 252530, 287533, 288052, 326336, 482084, 619536, 718821
85006.8	Micrococcales	12015, 16605, 33448, 35721, 50966, 51694, 53012, 57181, 195787, 218598, 241203, 253508, 265742, 273807, 276333, 278440, 297447, 297593, 297903, 298433, 315628, 316747, 321379, 516154, 540191, 540568, 592181, 616114, 619453, 1142462
85007.2	Mycobacteriales	1419, 4245, 4915, 9235, 10988, 11723, 16943, 19150, 35199, 44872, 51127, 51962, 92027, 183810, 186218, 194000, 199350, 279306, 294615, 427071, 431401, 467105, 467130, 467249, 475545, 503540, 540698, 565715, 641514, 1140293
85008.2	Micromonosporales	235, 32254, 182486, 186004, 198210, 198217, 198362, 198415, 199001, 199645, 248749, 283832, 285744, 295555, 478148, 482800, 542742, 546162, 590749, 598146, 599074, 608890, 612808, 612899, 622552, 649753, 652013, 653674, 680286, 681340
85009.2	Propionibacteriales	936, 50971, 51640, 184512, 187142, 188235, 194147, 198649, 198983, 199034, 199475, 216311, 275256, 279994, 288271, 291101, 293071, 293291, 294853, 295388, 308193, 313231, 325003, 326325, 523079, 527616, 530424, 616839, 662940, 683575
85010.0	Pseudonocardiales	7809, 33393, 62082, 184501, 185596, 186040, 198348, 199614, 204221, 238362, 249915, 272729, 282084, 292003, 294257, 294911, 295560, 295680, 321328, 323946, 501808, 533598, 535890, 552097, 569329, 580474, 597989, 660680, 1143463, 1165136
85011.4	Kitasatosporales	10931, 14390, 34196, 53024, 53127, 54804, 175829, 176087, 181951, 182206, 186318, 189065, 192930, 198750, 222531, 230407, 245372, 247634, 277533, 281963, 292452, 325849, 444212, 481022, 608955, 617734, 641386, 653493, 675554, 677896
85012.0	Streptosporangiales	31675, 198683, 198923, 198953, 215005, 238312, 249304, 253303, 294513, 295596, 306628, 309033, 316096, 501240, 523007, 534286, 576393, 579153, 579250, 583800, 586042, 605992, 614047, 628304, 640052, 644610, 646523, 660745, 669179, 1165074
85015.0	Nocardioidaceae	640, 3111, 50886, 50971, 51395, 51414, 51542, 51699, 51702, 77868, 194147, 198832, 199034, 199092, 230842, 244867, 246018, 275225, 276542, 293071, 293291, 307087, 308193, 309647, 500693, 530424, 549911, 640489, 662940, 663365
85016.0	Cellulomonadaceae	485, 849, 8460, 19753, 29839, 54314, 54319, 185663, 199404, 231693, 245142, 269289, 273256, 276333, 293764, 296469, 315994, 321181, 321386, 321484, 321720, 321723, 321798, 374843, 562124, 581206, 612875, 632740, 642125, 664209

Supplementary Table 4. (Continued.)

Set No.	Taxon name	Proteome IDs
85021.0	Intrasporangiaceae	5063, 8914, 13167, 19489, 19494, 30011, 30013, 35720, 35721, 51641, 76976, 175826, 182938, 199019, 233781, 237822, 278440, 316747, 317893, 319514, 319516, 321534, 321793, 431092, 437955, 554054, 573599, 588586, 592181, 677016
85023.3	Microbacteriaceae	24001, 33572, 33836, 37934, 50802, 50821, 51216, 51768, 51858, 69933, 190857, 195931, 199183, 228758, 238650, 243284, 243784, 270299, 274391, 275048, 298424, 321196, 422989, 501494, 515934, 517724, 573729, 610303, 636956, 1144396
85025.0	Nocardiaceae	4245, 6820, 8190, 13525, 19150, 19401, 76180, 76512, 178767, 183263, 188836, 199417, 215506, 219565, 249091, 255355, 277147, 283479, 292013, 295305, 432464, 438448, 503540, 515512, 516173, 535543, 620813, 638263, 640043, 654257
85030.0	Geodermatophilaceae	1382, 6461, 7517, 29713, 51830, 184471, 198403, 198589, 198952, 199152, 199500, 219482, 219514, 247602, 252403, 252630, 253027, 253175, 253272, 262362, 292507, 297607, 297781, 297836, 298197, 305560, 321490, 468828, 470470, 541969
91061.22	Bacilli	1279, 1651, 2895, 19474, 30147, 31546, 44136, 50929, 51658, 51878, 76382, 78148, 178622, 184128, 188246, 198714, 242949, 254924, 257055, 265711, 270219, 285120, 287101, 295257, 298347, 326671, 429595, 509424, 616608, 678895
91347.1	Enterobacterales	260, 815, 1123, 2084, 2529, 6859, 7841, 7966, 8148, 8216, 11834, 13568, 19025, 29462, 37088, 53401, 59419, 196435, 216612, 217182, 229786, 242222, 242496, 242642, 280899, 288794, 295719, 429602, 461443, 516181
92860.0	Pleosporales	1471, 16935, 16936, 53841, 77535, 193144, 266453, 676310, 756921, 758155, 799324, 799424, 799757, 799770, 800035, 800038, 800040, 800096, 813461, 1140562
92860.1	Pleosporales	2668, 76837, 77069, 77206, 193240, 240883, 596902, 651452, 700596, 730381, 799291, 799764, 799771, 799777, 800036, 800082, 800094, 801428, 1056012, 1140510
110618.0	Nectriaceae	5206, 9096, 16928, 37904, 91967, 219602, 241587, 266152, 544331, 547976, 558688, 582016, 593570, 622797, 730481, 737391, 750502, 813427, 1152087, 1152130
110618.1	Nectriaceae	7978, 16800, 30663, 287544, 288168, 532311, 536711, 546213, 554235, 567885, 569620, 604273, 626568, 694050, 717696, 720189, 736672, 738349, 782241, 813383
117743.1	Flavobacteriia	23541, 50827, 59672, 184231, 186230, 192360, 198379, 199439, 199595, 216840, 236641, 237310, 239068, 248536, 248840, 252687, 261828, 284892, 287527, 289734, 295215, 295468, 307602, 321094, 321938, 484164, 598120, 646211, 652681, 1139369
117747.1	Sphingobacteriia	28992, 31802, 186720, 192756, 192980, 198039, 198850, 215002, 236893, 240912, 244168, 244301, 245627, 260823, 283433, 284120, 286701, 291117, 293331, 294616, 295620, 307244, 326921, 451233, 472777, 505355, 515877, 556237, 614460, 646821
119045.0	Methylobacteriaceae	8207, 9081, 28825, 35489, 52050, 52062, 53043, 199229, 199569, 245444, 245926, 254925, 286997, 295122, 321085, 321258, 321605, 321750, 325614, 403266, 410984, 436483, 441523, 480288, 519439, 564885, 572984, 599312, 605848, 662825
119060.1	Burkholderiaceae	231, 605, 3511, 5045, 8815, 10322, 27020, 31838, 33618, 53474, 54717, 75613, 93802, 185151, 197215, 198866, 235347, 237381, 243719, 270342, 280434, 294982, 295382, 325273, 414233, 494363, 532440, 614287, 655523, 672934
135613.1	Chromatiales	1844, 1962, 2964, 5459, 9102, 17881, 19442, 31631, 34410, 34895, 66624, 189462, 192342, 199496, 199657, 218890, 223759, 232638, 243679, 245474, 252707, 276260, 295707, 295717, 297890, 325372, 426424, 433788, 438795, 548632
135614.1	Xanthomonadales	1010, 4210, 29391, 29708, 33067, 50940, 51386, 51430, 182005, 188464, 198575, 198725, 199420, 245812, 251842, 253740, 254438, 274358, 291286, 291562, 308707, 315167, 315271, 315891, 319980, 462066, 543279, 555859, 639274, 1139971
135619.1	Oceanospirillales	238, 239, 1062, 6286, 14463, 28073, 78070, 182350, 185639, 186895, 187327, 190064, 198500, 198641, 199677, 244044, 254326, 257039, 267342, 276016, 283087, 287023, 317839, 327197, 518892, 547614, 565262, 595332, 599578, 1058168
135620.0	Oceanospirillaceae	11866, 27318, 32749, 33747, 54058, 78070, 92544, 92627, 186895, 191418, 199058, 202440, 236745, 242469, 242999, 250744, 253769, 256542, 282818, 283087, 324760, 325302, 502243, 538931, 565262, 595663, 599578, 628710, 640333, 1150830
135621.1	Pseudomonadaceae	2515, 15503, 16560, 29493, 30063, 32531, 33462, 51864, 56648, 196445, 198706, 199524, 232228, 243063, 243207, 243232, 243299, 243488, 243778, 243924, 244119, 251762, 270661, 278332, 294575, 306753, 315235, 319627, 327522, 509591
135622.1	Alteromonadales	1558, 1982, 2608, 6327, 6334, 6843, 14461, 16487, 19276, 33452, 53586, 70299, 194841, 219285, 244441, 247426, 256561, 286482, 294826, 297642, 304912, 307702, 316850, 327086, 481517, 502608, 601768, 606935, 623842, 682739
135623.0	Vibrionales	593, 2493, 2943, 3604, 16562, 16570, 16895, 29994, 31672, 32303, 33633, 37515, 51221, 92876, 94070, 94165, 94936, 184608, 188276, 193432, 198854, 215148, 219336, 235828, 281112, 462621, 464262, 533102, 535589, 1155586
147541.0	Dothideomycetes	8062, 11761, 16931, 16933, 75602, 183809, 194280, 225277, 266453, 676310, 700596, 756921, 785799, 799324, 799757, 799777, 800035, 800040, 801428, 809789
147541.1	Dothideomycetes	30672, 30706, 33647, 77206, 215127, 240883, 243723, 298493, 325902, 504637, 758155, 799424, 799436, 799750, 799767, 800092, 800094, 800235, 813461, 1056012

Supplementary Table 4. (Continued.)

Set No.	Taxon name	Proteome IDs
147541.2	Dothideomycetes	1067, 1471, 2668, 16934, 16936, 53259, 53841, 77069, 237631, 243797, 308549, 310066, 316270, 660729, 799423, 799429, 799439, 799441, 799770, 1140510
147541.3	Dothideomycetes	27730, 70133, 76837, 192596, 309340, 504636, 596902, 651452, 714618, 730381, 799437, 799438, 799537, 799771, 800036, 800038, 800039, 800041, 800082, 1140562
147545.0	Eurotiomycetes	1261, 2530, 7304, 19471, 24533, 42958, 53328, 53599, 184304, 184546, 191612, 224080, 246702, 248961, 249363, 325672, 606974, 661057, 1149074, 1150879
147545.1	Eurotiomycetes	560, 2669, 6702, 6706, 19478, 36893, 55045, 70168, 191342, 191408, 214365, 247810, 256690, 286921, 326565, 326950, 1141434, 1147782, 1149163, 1150904
147545.2	Eurotiomycetes	724, 1294, 1745, 2038, 2497, 54342, 191518, 226031, 234275, 234585, 247233, 248340, 248349, 326289, 452235, 1147747, 1148299, 1149165, 1150941, 1153618
147545.3	Eurotiomycetes	19473, 27920, 53411, 94569, 184188, 191672, 218381, 223968, 231358, 248423, 249402, 249829, 253729, 326198, 509510, 631181, 637239, 1146351, 1147752, 1154252
147545.4	Eurotiomycetes	8142, 19484, 34291, 37505, 53342, 54302, 177622, 182235, 184300, 184499, 186955, 215305, 242791, 243015, 319663, 325780, 326532, 326799, 541154, 641853
147545.5	Eurotiomycetes	1631, 2035, 9882, 18001, 19804, 53617, 54383, 54466, 78343, 179179, 184073, 184383, 188318, 215289, 327118, 701341, 1147733, 1149079, 1149954, 1152649
147545.6	Eurotiomycetes	6701, 19376, 30104, 30143, 37696, 54771, 94526, 184356, 190312, 191285, 191691, 234474, 325579, 326268, 326931, 653565, 654913, 1147746, 1147760, 1150942
147548.0	Leotiomycetes	1798, 6753, 15441, 16922, 178129, 184330, 235371, 241818, 242519, 256645, 283383, 286134, 297299, 297527, 326757, 531561, 624404, 696280, 701801, 1152300
147550.0	Sordariomycetes	5206, 5426, 7322, 8782, 11096, 37136, 76580, 78544, 237481, 242955, 275385, 319160, 326340, 544331, 558688, 694050, 699042, 717696, 720189, 813383
147550.1	Sordariomycetes	1197, 9058, 9096, 16928, 27238, 36947, 54544, 70121, 76744, 194361, 286045, 288168, 532311, 605986, 654918, 722485, 749293, 756346, 1140502, 1152049
147550.2	Sordariomycetes	2762, 6039, 7115, 8066, 9097, 18087, 30651, 54821, 193689, 233524, 241690, 285146, 305883, 481858, 515153, 536711, 547976, 696573, 738349, 1152087
147550.3	Sordariomycetes	2499, 34112, 76552, 78397, 78576, 91967, 222788, 236621, 240493, 241462, 243732, 266152, 283895, 297716, 557566, 567885, 574317, 635477, 827724, 829364
147550.4	Sordariomycetes	1610, 30753, 70720, 76874, 94444, 226192, 235728, 236664, 241587, 265631, 266234, 287144, 310108, 315783, 604273, 622797, 626568, 736672, 781932, 1152130
147550.5	Sordariomycetes	1881, 7978, 8513, 14480, 15530, 16800, 78340, 245910, 266272, 277212, 287544, 325492, 434172, 554235, 562929, 569620, 593570, 777438, 811619, 824596
147550.6	Sordariomycetes	8181, 8984, 16801, 16923, 28524, 37904, 54516, 76584, 76881, 295703, 517252, 582016, 639643, 730481, 750502, 782241, 813426, 813427, 813444, 830671
147550.7	Sordariomycetes	1805, 7796, 20467, 27002, 30106, 30663, 30816, 31176, 70501, 176998, 182658, 186583, 241546, 243498, 252748, 284375, 546213, 737391, 748025, 758603
155619.0	Agaricomycetes	27222, 53257, 54097, 54166, 54477, 54485, 54538, 54988, 217199, 217790, 219338, 230002, 283269, 287166, 294933, 623687, 629468, 772434, 807306, 813600
155619.1	Agaricomycetes	17559, 27073, 27195, 27265, 53558, 53712, 53989, 76761, 76871, 77086, 193067, 284706, 313359, 521943, 664032, 736335, 799118, 807469, 1049176, 1142393
155619.2	Agaricomycetes	1861, 7431, 8493, 30669, 53477, 54018, 76154, 92154, 184267, 194127, 218811, 250043, 305948, 307440, 320762, 322482, 365756, 703269, 823399, 1150266
155619.3	Agaricomycetes	6352, 16930, 30671, 54007, 76532, 76727, 77266, 218334, 242287, 292082, 298030, 305067, 308652, 521872, 609673, 714275, 724874, 807342, 807769, 812287
165695.0	*Sphingobium*	1275, 7753, 9887, 13201, 15523, 15524, 18867, 24284, 52232, 56968, 60779, 78304, 78322, 94323, 199142, 218272, 239825, 267239, 279959, 282977, 290958, 290975, 426201, 549617, 552700, 552757, 571950, 576821, 681425, 1138757
165696.0	*Novosphingobium*	4030, 4728, 7515, 9134, 9242, 37998, 50893, 52268, 56630, 58012, 61032, 199304, 220675, 232587, 236327, 295319, 321172, 323719, 426325, 431236, 465810, 522081, 548867, 551327, 555448, 562395, 566813, 608154, 617634, 648075
171549.1	Bacteroidales	3089, 3167, 5436, 5580, 5697, 15993, 17831, 18054, 18057, 18062, 18111, 18392, 18727, 23482, 29597, 30116, 30150, 56419, 95517, 177035, 195772, 199452, 216094, 235918, 251835, 297225, 324383, 500961, 555103, 629836
171552.0	Prevotellaceae	2772, 2786, 3112, 3160, 3327, 3460, 3829, 4477, 5141, 5580, 10433, 10460, 10862, 15929, 15993, 16600, 17958, 18116, 18343, 18353, 18359, 18375, 70533, 198673, 198779, 199132, 216094, 216769, 272376, 825483
186801.13	Clostridia	3244, 5561, 8234, 18023, 18124, 29585, 31366, 32483, 35704, 50326, 175744, 182321, 182569, 184342, 184869, 190657, 196526, 198636, 199228, 199394, 242329, 243547, 261080, 271256, 287890, 294919, 503601, 515703, 617951, 657006
186802.6	Eubacteriales	4073, 4754, 8937, 10847, 14108, 17961, 18037, 18082, 18137, 19482, 29585, 29916, 36923, 76603, 78233, 184080, 184447, 186102, 195869, 198597, 220840, 236356, 264338, 287890, 298460, 323166, 474718, 597668, 651482, 661435

Supplementary Table 4. (Continued.)

Set No.	Taxon name	Proteome IDs
186803.3	Lachnospiraceae	4121, 5396, 5561, 8178, 17921, 18002, 18056, 18163, 18690, 95409, 183918, 184038, 186530, 195974, 196053, 198806, 198838, 199418, 199659, 199701, 199786, 242090, 245488, 250003, 260812, 292927, 295711, 652477, 655830, 712157
186806.0	Eubacteriaceae	4754, 5178, 12589, 14176, 17904, 17940, 17954, 17996, 18124, 18312, 18318, 18404, 18411, 18414, 36873, 184251, 189857, 190657, 190814, 196365, 198817, 199228, 199394, 253490, 262191, 284400, 284779, 440004, 616595, 663499
186817.5	Bacillaceae	594, 6316, 11747, 27980, 30401, 54099, 74108, 180199, 193006, 198778, 199066, 199668, 216498, 226441, 247922, 263943, 267430, 270219, 271374, 276349, 297982, 325032, 615181, 637720, 660110, 666543, 678477, 678491, 1084197, 1145069
186818.0	Planococcaceae	6691, 11919, 19062, 30408, 30416, 31496, 31938, 58129, 67683, 70230, 93199, 188184, 217065, 222866, 245938, 251002, 254519, 265725, 271255, 275473, 288623, 295327, 297458, 315753, 316502, 321901, 473875, 622653, 658225, 680589
186822.1	Paenibacillaceae	1877, 3445, 29487, 29512, 36611, 37269, 61660, 77355, 78148, 93309, 93585, 187172, 193834, 195591, 198936, 215509, 215741, 247476, 266482, 272464, 289396, 298246, 316330, 317036, 321157, 478765, 574133, 632125, 650466, 677034
186826.0	Lactobacillales	432, 449, 2707, 5147, 7946, 8129, 12675, 16057, 18559, 32287, 51296, 51450, 51679, 51804, 52013, 70387, 182149, 182508, 187499, 191200, 195918, 198374, 198833, 234775, 242754, 279194, 287857, 288197, 310506, 1139006
194924.0	Desulfovibrionaceae	2194, 2601, 2710, 4662, 6034, 6424, 11724, 14960, 14975, 16587, 91979, 181901, 186323, 186469, 189733, 190027, 198324, 218812, 219215, 266086, 267796, 269883, 438699, 448292, 461162, 469724, 473968, 494245, 503820, 580856
200643.4	Bacteroidia	3167, 3327, 3460, 4156, 4477, 4524, 5697, 10460, 18026, 18121, 18343, 18392, 30103, 30150, 76586, 95517, 177035, 184436, 184509, 191055, 196154, 198779, 199132, 215155, 243574, 249300, 255233, 279562, 282985, 297225
200644.6	Flavobacteriales	1602, 4690, 5638, 5938, 6650, 76715, 93807, 184225, 192393, 195822, 198384, 198792, 199448, 199595, 231960, 232401, 233435, 238805, 247903, 249542, 254305, 261828, 321080, 321954, 443153, 484164, 608754, 651057, 656244, 712080
200666.1	Sphingobacteriales	852, 2774, 28992, 31802, 50543, 62859, 95113, 178216, 189739, 189981, 192756, 199577, 199666, 238642, 244168, 245627, 248198, 284120, 291117, 294616, 295499, 295668, 310477, 320042, 325105, 325329, 521691, 646821, 651668, 1139450
200940.1	Thermodesulfobacteriota	1933, 2420, 2534, 2710, 5778, 6365, 6424, 6583, 8561, 14975, 14977, 16587, 68196, 75994, 182264, 191931, 198771, 199355, 199602, 218812, 269883, 317155, 318307, 324159, 422108, 494220, 525298, 596092, 663722, 1144372
201174.32	Actinomycetota	4830, 14204, 15001, 28870, 34098, 35034, 51640, 95759, 192775, 199323, 221369, 270471, 277858, 292547, 321196, 325988, 326179, 463027, 466517, 467328, 503297, 521922, 540423, 557204, 614239, 617734, 619355, 619453, 654257, 1138997
203682.1	Planctomycetota	1887, 6860, 11885, 11991, 76318, 76398, 187735, 199518, 245802, 280296, 315017, 315440, 315724, 317238, 318017, 318053, 318478, 318741, 318878, 318995, 320496, 322699, 324233, 324479, 324974, 326837, 464378, 551616, 676194, 1155241
203683.0	Planctomycetia	1887, 6860, 76398, 187735, 199518, 245802, 280296, 315017, 315440, 315471, 315700, 316095, 316598, 317421, 317648, 317835, 318017, 318053, 318437, 318538, 318995, 319976, 320496, 320672, 322214, 324974, 464378, 503447, 551616, 1155241
203691.0	Spirochaetota	332, 503, 811, 1408, 1847, 2967, 3571, 5737, 6546, 6852, 7383, 8212, 9222, 9223, 14541, 18680, 42527, 182360, 190423, 253869, 294625, 297855, 298058, 323824, 324209, 515827, 518887, 578697, 587760, 660053
203692.0	Spirochaetia	1807, 2318, 2967, 5737, 6048, 6546, 7254, 7383, 9222, 14541, 14605, 15620, 18680, 182360, 186400, 190395, 190423, 192343, 245133, 294625, 297855, 298058, 323824, 324209, 324638, 515827, 518887, 578697, 587760, 671908
204432.0	Terriglobia	343, 2207, 2432, 6056, 6844, 7113, 182427, 198356, 199024, 236728, 253606, 264702, 269669, 289437, 290253, 292958, 295210, 321820, 513480, 515312, 515460, 515496, 538666, 540989, 568106, 589520, 593892, 647241, 648801, 1059380
204433.0	Terriglobales	343, 2207, 2432, 6056, 6844, 7113, 182427, 198356, 199024, 236728, 253606, 264702, 269669, 279598, 289437, 290253, 292958, 295210, 321820, 513480, 515312, 515460, 515496, 538666, 540989, 568106, 589520, 647241, 648801, 1059380
204441.3	Rhodospirillales	245, 7058, 32356, 179145, 185678, 186308, 216998, 231658, 237344, 239724, 245629, 245765, 247565, 277007, 280346, 295023, 295096, 305654, 315252, 317730, 321405, 321746, 553706, 580654, 581135, 600101, 631034, 677537, 761264, 830341
204455.3	Rhodobacterales	2931, 3635, 6762, 18782, 23430, 27746, 31326, 176562, 184932, 192273, 193409, 198307, 198761, 199180, 199344, 202485, 215377, 238338, 244924, 248916, 295142, 309747, 322545, 434754, 436694, 468591, 477083, 526408, 609121, 639775
204457.4	Sphingomonadales	9134, 11717, 11816, 13072, 18851, 27647, 33202, 37930, 55668, 78322, 195807, 198359, 219494, 236327, 254856, 309389, 320547, 325427, 431236, 445582, 466966, 471147, 471435, 509259, 516148, 522753, 538147, 546200, 575068, 584526

Supplementary Table 4. (Continued.)

Set No.	Taxon name	Proteome IDs
204458.0	Caulobacterales	1364, 1492, 1816, 1868, 2696, 17808, 17812, 17826, 17837, 37838, 50923, 51150, 51182, 51604, 56905, 195546, 228945, 244913, 247763, 249524, 249725, 249842, 317223, 431269, 482044, 548978, 622580, 662572, 663918, 676409
206351.0	Neisseriales	1274, 1424, 2010, 3009, 3019, 4088, 4105, 4207, 17813, 18554, 31390, 36114, 76625, 188659, 198213, 215450, 219669, 230202, 242869, 244173, 254293, 254651, 282438, 310016, 325536, 504844, 509597, 543030, 645257, 653156
206389.0	Rhodocyclales	2186, 6552, 13140, 70186, 186819, 187526, 198607, 215181, 217984, 234247, 241193, 241885, 242205, 244972, 248259, 257259, 295129, 307956, 308430, 318422, 323671, 389128, 463961, 561045, 580043, 587070, 599523, 663379, 663444, 694660
213115.0	Desulfovibrionales	1052, 2194, 2216, 2601, 4662, 6424, 7844, 14960, 14975, 16587, 91979, 95200, 181901, 186323, 189733, 190027, 198324, 198771, 199355, 199602, 218812, 219215, 240513, 267796, 269883, 448292, 461162, 469724, 494245, 503820
213849.0	Campylobacterales	939, 2495, 5085, 5709, 7032, 8633, 8721, 15520, 29920, 34444, 92884, 186074, 194309, 201169, 230780, 249746, 251062, 251135, 251881, 255139, 256421, 256695, 257067, 319322, 326944, 476338, 502820, 503264, 509414, 671852
216572.1	Oscillospiraceae	2145, 3438, 4259, 14129, 18037, 18181, 18315, 18328, 19365, 29585, 196146, 196191, 196537, 199158, 233534, 253465, 264338, 276301, 285063, 301475, 306409, 324781, 469514, 474718, 597668, 610760, 620327, 660861, 661435, 774750
335929.0	Erythrobacteraceae	2995, 16568, 49978, 53070, 53455, 77326, 78263, 92932, 275232, 284395, 309389, 315722, 320547, 325427, 326213, 429229, 431922, 433104, 433652, 439522, 445582, 448199, 460561, 461409, 469159, 469430, 473531, 502110, 546031, 594459
423349.0	*Mucilaginibacter*	2774, 189739, 199072, 199679, 215002, 242687, 244168, 245678, 253209, 260823, 268007, 282759, 286701, 293331, 318733, 320042, 321479, 429232, 503278, 505355, 521691, 538158, 556237, 613193, 619078, 622475, 638732, 646821, 662074, 1139450
563835.0	Chitinophagaceae	3586, 31408, 33121, 77177, 77667, 184420, 192276, 192610, 192796, 198711, 199031, 199537, 217148, 239872, 240572, 240971, 242818, 244450, 249720, 263900, 267223, 279089, 281028, 290204, 305848, 321204, 321513, 321533, 326903, 1155483
681950.0	Glomerellaceae	8782, 11096, 14480, 15530, 20467, 27238, 70121, 76552, 176998, 186583, 295703, 305883, 326340, 434172, 613401, 639643, 654918, 699042, 781932, 830671
768503.1	Cytophagia	493, 2011, 10796, 50454, 59956, 61382, 75267, 75606, 189843, 192266, 193660, 198432, 199437, 199513, 234203, 245468, 251692, 295706, 297549, 297647, 322791, 441336, 480178, 501128, 509425, 515410, 537126, 598820, 603640, 609064
768507.1	Cytophagales	2011, 9309, 10953, 19423, 30789, 50454, 51810, 61382, 75583, 184212, 184609, 192266, 192333, 194873, 198432, 198510, 199306, 245468, 251692, 297549, 305398, 317847, 478546, 501623, 502756, 515410, 603640, 612233, 632594, 664144
909932.0	Negativicutes	1902, 3195, 3277, 3503, 4923, 7093, 9883, 10111, 11124, 14380, 14944, 16614, 29628, 36503, 41641, 95546, 182958, 195529, 198896, 199309, 199689, 215383, 276437, 277811, 284368, 295063, 295188, 320776, 323646, 591941
1028384.0	Glomerellales	8782, 11096, 14480, 15530, 20467, 27238, 70121, 76552, 76584, 176998, 186583, 295703, 305883, 326340, 434172, 613401, 639643, 699042, 781932, 830671
1131492.0	Aspergillaceae	9882, 19804, 70168, 177622, 179179, 184188, 184546, 215289, 218381, 246702, 247233, 248349, 248423, 325579, 326289, 452235, 1146351, 1147760, 1147782, 1149954
1131492.1	Aspergillaceae	2530, 6702, 19376, 34291, 37696, 94569, 184300, 184304, 184383, 186955, 188318, 190312, 191285, 248961, 249402, 325780, 326950, 327118, 631181, 1149163
1131492.2	Aspergillaceae	6701, 30104, 36893, 37505, 42958, 54771, 191408, 231358, 234474, 248340, 256690, 319663, 1147733, 1147746, 1148299, 1149074, 1150879, 1150941, 1150942, 1152649
1131492.3	Aspergillaceae	560, 30143, 184356, 184499, 215305, 234275, 247810, 253729, 326198, 326268, 326532, 326799, 326931, 630445, 637239, 641853, 1147747, 1150904, 1153618, 1154252
1131492.4	Aspergillaceae	724, 6706, 55045, 184073, 191342, 191518, 191612, 191672, 234585, 286921, 325672, 326565, 541154, 653565, 654913, 661057, 701341, 1147752, 1149079, 1149165
1643682.0	Geodermatophilales	1382, 6461, 7517, 29713, 51830, 184471, 198403, 198589, 198857, 198952, 199152, 199500, 219482, 219514, 237752, 252403, 252630, 253027, 253175, 253272, 262362, 292507, 297607, 297781, 298197, 305560, 321490, 468828, 470470, 541969
1706369.0	Cellvibrionales	1036, 1947, 4699, 9080, 29640, 76077, 77184, 188219, 193450, 196143, 234845, 267187, 273643, 275394, 294980, 295554, 319732, 326835, 467441, 528457, 535937, 539350, 559987, 596063, 610558, 652567, 664303, 787472, 1056808, 1069090
1737404.0	Tissierellia	1319, 3280, 3422, 3705, 4191, 4712, 5286, 6094, 17105, 31386, 32409, 37267, 70442, 179042, 180254, 184032, 184114, 184389, 186112, 198828, 245423, 261011, 269544, 284177, 294567, 297454, 298381, 377798, 1108123, 1142078
1737405.0	Tissierellales	1319, 3280, 3422, 3705, 3821, 4191, 4712, 5984, 6094, 13378, 17105, 31386, 32409, 37267, 70442, 179042, 180254, 184032, 184114, 184389, 186112, 198828, 245423, 261011, 294567, 297454, 298381, 377798, 1108123, 1142078

Supplementary Table 4. (Continued.)

Set No.	Taxon name	Proteome IDs
1775411.0	Rhodanobacteraceae	3226, 5234, 29708, 76131, 182005, 184078, 188464, 189228, 198575, 198725, 199420, 199603, 235676, 241074, 245812, 253740, 267077, 270530, 288967, 291822, 294599, 295293, 306317, 307749, 490980, 521199, 543279, 555859, 667269, 1139971
1822464.0	*Paraburkholderia*	1192, 3511, 5045, 30460, 75613, 76852, 93802, 185151, 187012, 198638, 198866, 199120, 199548, 237381, 254875, 263097, 272778, 294982, 325273, 433577, 434209, 494119, 494252, 494255, 494329, 494363, 494365, 655523, 676887, 1139308
1853228.1	Chitinophagia	31408, 77667, 184368, 184420, 185003, 186917, 190166, 190888, 192796, 198757, 199041, 217148, 239872, 240572, 242818, 249547, 253410, 266118, 279089, 281028, 290204, 290545, 292424, 293874, 305848, 321436, 321513, 326903, 476411, 627292
1853229.1	Chitinophagales	3586, 31400, 33121, 77177, 184420, 188534, 190166, 190888, 192276, 192610, 198757, 199041, 199537, 217148, 244333, 244450, 248745, 249720, 266118, 279089, 290204, 293874, 294498, 305848, 321204, 321533, 476411, 569858, 598971, 607559
1853232.0	Hymenobacteraceae	19423, 28590, 30789, 33109, 36458, 59542, 59956, 70672, 182491, 184418, 185924, 189843, 192266, 198432, 198697, 251692, 253919, 262802, 321532, 321926, 322791, 326570, 441336, 478546, 515410, 559626, 563094, 603640, 632594, 664144
1866885.0	*Mycolicibacterium*	757, 4915, 5442, 6057, 6265, 9159, 18763, 28870, 32221, 36176, 57134, 70612, 94243, 178953, 192801, 193484, 193564, 220914, 254978, 279306, 282551, 323700, 325690, 466445, 466517, 466931, 467006, 467193, 467249, 1140293
1890424.0	Synechococcales	788, 1115, 1420, 1422, 1423, 1425, 1430, 1566, 1938, 1961, 2535, 3950, 4972, 5766, 10379, 10385, 10482, 28237, 28591, 76867, 182608, 238762, 240206, 243002, 325996, 515370, 594525, 594557, 607397, 889800
1913637.0	Mucoromycota	14254, 18888, 27586, 53815, 77051, 77315, 93000, 242146, 242381, 247702, 252139, 266673, 646827, 654370, 702732, 707451, 726737, 740926, 764092, 1153678
1913637.1	Mucoromycota	54107, 78512, 78561, 193560, 193648, 234323, 242180, 242254, 263633, 265703, 439903, 603453, 650833, 696485, 716291, 723463, 738325, 748756, 764349, 807716
2691354.0	Pirellulales	1025, 11885, 11991, 76398, 315440, 315471, 316213, 316304, 316598, 316770, 317421, 317429, 317648, 317909, 317977, 318053, 318288, 318437, 318538, 318878, 318995, 319143, 319852, 320176, 321353, 322699, 324479, 325286, 536179, 551616
2726947.0	Mycosphaerellales	8062, 16931, 16932, 16933, 33647, 70133, 75602, 215127, 225277, 237631, 308549, 309340, 310066, 504637, 660729, 756132, 799537, 799539, 799767, 1056384
2762318.0	Weeksellaceae	6051, 6085, 6276, 8641, 28641, 35900, 92708, 95601, 182761, 184335, 188947, 191112, 192393, 198517, 198931, 215196, 238042, 252172, 270538, 273471, 279525, 294419, 309120, 464318, 552241, 553459, 608754, 610746, 694480, 1152599
2854170.3	Roseobacteraceae	2944, 5713, 19593, 26249, 31171, 31326, 48908, 184221, 192455, 193409, 193570, 198634, 198728, 198767, 198994, 199026, 199283, 199550, 199628, 202922, 203589, 244912, 252940, 274386, 284407, 293145, 305887, 325526, 327025, 503308
2887326.0	Moraxellales	430, 546, 5740, 13117, 13148, 14568, 18415, 18418, 23785, 23788, 23795, 35860, 64939, 76238, 92616, 92671, 93391, 185895, 187495, 188357, 189800, 190435, 196536, 242317, 243900, 244223, 245977, 253940, 255230, 256774
2975441.0	Sphaerotilaceae	366, 1693, 7883, 53102, 56576, 60699, 193427, 197446, 197468, 199246, 238605, 239406, 247811, 274884, 288178, 288587, 293433, 295110, 295361, 301751, 323522, 430120, 484255, 518288, 529637, 554837, 574067, 594526, 620139, 678374
3028117.0	Cyanophyceae	268, 1115, 1191, 1203, 1566, 5766, 10383, 10384, 10386, 10390, 10477, 10483, 16960, 28591, 29738, 182608, 215660, 218785, 243002, 252107, 271624, 282767, 503129, 596184, 607678, 619131, 627856, 627988, 632766, 645501
3031449.0	Desulfovibrionia	1052, 2194, 2216, 2601, 4662, 5496, 6424, 7844, 11724, 14975, 16587, 91979, 95200, 181901, 186323, 186469, 189733, 190027, 198771, 199355, 199602, 218812, 240513, 267796, 269883, 438699, 448292, 461162, 469724, 473968
3031852.0	Epsilonproteobacteria	422, 760, 1136, 2222, 2407, 5085, 5709, 6431, 7032, 7727, 7803, 8387, 8633, 14539, 18688, 29313, 194309, 229442, 230780, 251062, 251881, 254920, 256421, 256695, 257067, 272365, 451105, 476338, 503264, 509414
3082720.0	Peptostreptococcales	269, 3178, 3244, 5244, 17980, 19591, 31189, 34407, 93352, 184082, 184465, 190140, 190285, 198304, 198718, 199208, 199230, 199568, 215694, 216024, 242520, 243255, 243494, 245695, 284841, 287601, 294919, 295500, 295504, 503601
3085636.3	Lachnospirales	370, 1476, 17909, 18002, 37233, 95380, 95409, 183918, 184301, 198508, 198838, 199315, 199800, 199802, 199820, 236497, 245488, 280696, 285302, 290106, 292927, 295711, 298653, 460412, 464314, 483018, 543642, 623269, 677305, 712157
